# Dioecious *Poa* species diversification parallels dune formation in Atlantic coastal ecosystems

**DOI:** 10.1093/aobpla/plag028

**Published:** 2026-06-08

**Authors:** Liliana Mónica Giussani, Agostina Belén Sassone, Florencia Rocío Sabena, Cintia Eleonora Celsi, Franco Santin, Leopoldo Javier Iannone

**Affiliations:** Instituto de Botánica Darwinion (IBODA – ANCEFN – CONICET), Labardén 200, San Isidro, Buenos Aires B1642HYD, Argentina; Leibniz Institute of Plant Genetics and Crop Plant Research (IPK), Corrensstraße 3, D-06466, OT Gatersleben, Germany; Instituto de Botánica Darwinion (IBODA – ANCEFN – CONICET), Labardén 200, San Isidro, Buenos Aires B1642HYD, Argentina; Instituto de Micología y Botánica (INMIBO – CONICET) - Universidad de Buenos Aires, Intendente Güiraldes 2160, Ciudad Autónoma de Buenos Aires C1428EGA, Argentina; Departamento de Biodiversidad y Biología Experimental, Facultad de Ciencias Exactas y Naturales, Universidad de Buenos Aires, Intendente Güiraldes 2160, Ciudad Autónoma de Buenos Aires C1428EGA, Argentina; Fundación de Historia Natural Félix de Azara, Hidalgo 775, Ciudad Autónoma de Buenos Aires C1425BCK, Argentina; Instituto de Investigaciones en Ingeniería Genética y Biología Molecular ‘Dr. Héctor N. Torres’ (INGEBI – CONICET), Vuelta de Obligado 2490, Ciudad Autónoma de Buenos Aires C1428ADN, Argentina; Instituto de Micología y Botánica (INMIBO – CONICET) - Universidad de Buenos Aires, Intendente Güiraldes 2160, Ciudad Autónoma de Buenos Aires C1428EGA, Argentina; Departamento de Biodiversidad y Biología Experimental, Facultad de Ciencias Exactas y Naturales, Universidad de Buenos Aires, Intendente Güiraldes 2160, Ciudad Autónoma de Buenos Aires C1428EGA, Argentina

**Keywords:** *Dioicopoa*, coastal dunes, genotypic diversity, phylogenomics, morphological differentiation, endemism, *Poa schizantha*, *Poa bergii*, introgression, conservation

## Abstract

The southern Atlantic coastal dunes of South America formed ca. 6000–4000 years ago. Within this dynamic landscape, three dioecious species of *Poa* occur: *Poa lanuginosa*, a widely distributed species, and two dune endemics, *Poa bergii* and *Poa schizantha*, occupying contrasting microhabitats from foredunes to interdunal slacks and inland grasslands. We investigated the evolutionary relationships, population structure, and extent of introgression among these dune-adapted grasses, conducting a population-level phylogenomic analysis using genotyping-by-sequencing. Genome-wide data provided robust phylogenetic resolution, whereas Neighbor-Net analyses revealed reticulate relationships and admixture among taxa. We tested introgression, and significant signals of gene flow were detected across major groups. Bayesian and discriminant analysis of principal components analyses identified distinct genetic lineages within both dune endemics. *Poa bergii* comprised three geographically structured genetic groups, and *P. schizantha* showed clear internal differentiation despite its restricted distribution. Instead, *P. lanuginosa* has a broad distribution across both dune and continental habitats and was inferred to be a paraphyletic lineage that may represent the ancestral lineage of both dune endemic species. These distinct patterns of divergence and population structure indicate species-specific evolutionary responses to a recent and dynamic dune landscape, in which habitat heterogeneity and differential environmental tolerance have driven lineage diversification. Together, our findings highlight the role of coastal dune dynamics in recent adaptation and speciation. Ongoing gene flow alongside clear lineage structure is consistent with recent and still incomplete speciation among these dune species. *Poa schizantha* emerged as the most differentiated lineage, combining morphological distinctiveness, ecological specialization, and strong genomic structure. Our results emphasize the importance of integrating population genetics into conservation strategies to preserve both endemic taxa and ongoing evolutionary processes in threatened coastal environments.

## Teaser

The southern Atlantic coastal dunes form a dynamic and recent landscape shaped only 6000 years ago. Using population-level phylogenomics, we investigated how habitat heterogeneity promoted evolutionary divergence within this system. Our results reveal recent and still incomplete differentiation: despite ongoing gene flow, well-defined genetic lineages persist. Among them, both endemic dune species, *Poa schizantha* and *P. bergii*, likely diversified from *P. lanuginosa* through ecological specialization. This study not only clarifies how coastal dynamics foster adaptation and speciation but also highlights the importance of incorporating population genetics into conservation strategies aimed at preserving these ongoing evolutionary processes in threatened dune environments.

## Introduction

Along the South American Atlantic coast, dune systems form a continental strip parallel to the ocean, occurring as discontinuous patches of variable width (0.3–15 km) throughout the Atlantic coasts of Brazil, Uruguay, and Argentina (López and Marcomini 2011). These coastal dunes constitute fragile ecosystems where plants are exposed to multiple environmental stresses, including high levels of solar radiation, strong winds, salinity, unstable sandy substrate, and wide diurnal temperature fluctuations (Hesp 1991, Celsi and Monserrat 2008, Celsi 2016). Within this system, fixed dune grasslands and shrublands occur inland farther from the shoreline, beyond the foredunes and active dune zone (Monserrat *et al.* 2012, Scherer *et al.* 2020, Toffani *et al.* 2024). The foredunes and active dune zones are typically dominated by grasses of the genera *Panicum* L., *Poa* L., and *Sporobolus* R.Br., together with Asteraceae species of the genera *Baccharis* L., *Hyalis* D. Don ex Hook. & Arn., and *Senecio* L., among other herbaceous taxa (Hesp 2002, Celsi and Monserrat 2008, Sloss *et al.* 2012, Celsi and Giussani 2020). These species play a crucial role in soil stabilization and facilitate the establishment of other species. As early colonizers, they are fundamental to vegetation succession, promoting the development of different plant community assemblages (Rivera *et al.* 2025).

**Figure 1:**
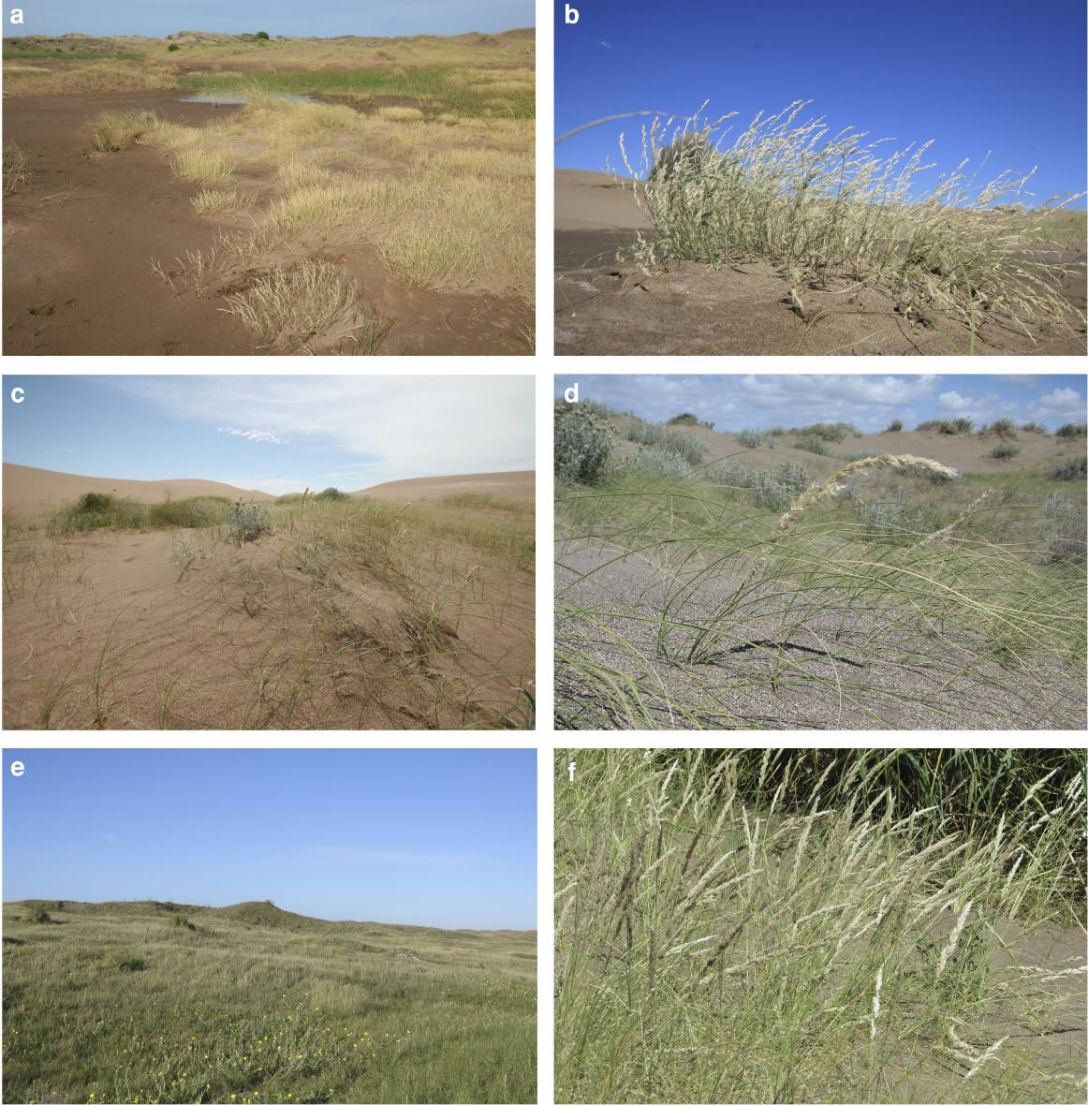
Habitat landscapes (left panels) and plant growth habit (right panels) of coastal dune *Poa* species. The ecological niches of the three species are partially segregated across coastal dune habitats. (a) Interdunal slack habitat and (b) growth habit of *P. schizantha*. (c) Active dune field and (d) growth habit of *P. bergii*. (e) Fixed dune grassland and (f) growth habit of *P. lanuginosa*.

Several dioecious *Poa* species of sects. *Madropoa* and *Dioicopoa* inhabit coastal dunes throughout the Americas. Within *Dioicopoa*, species and species complexes are distributed in geographically restricted regions of South America, suggesting an evolutionary pattern shaped by habitat specialization (Giussani 2000). The dioecious Atlantic coastal species—*Poa bergii* Hieron., *Poa lanuginosa* Poir., and *Poa schizantha* Parodi—provide an illustrative example of how habitat specialization has shaped species divergence. *Poa lanuginosa* is a widely distributed native species of arid and sandy environments south of 36°S, occurring in both coastal dunes and inland continental habitats in Pampean and Patagonian regions (Giussani *et al.* 2012). In contrast, the other two species are endemic to southern Atlantic coastal dunes: *P. bergii* extends from Buenos Aires to Chubut provinces, whereas *P. schizantha* is narrowly endemic, being confined to a ∼51km stretch of coastline, restricted to the dunes of Coronel Dorrego and Monte Hermoso localities in southern coast of Buenos Aires province, Argentina (Celsi and Giussani 2020). Although all three species occur within dynamic dune fields, they are associated with different habitats (Fig. 1). Species ecological niches are partially segregated. *Poa bergii* and *P. lanuginosa* are found in the foredune, an area eventually subject to the advance and retreat of the sea, as well as in the active dune zone, where they are exposed to shifting sand (Sabena 2019). *Poa lanuginosa* is also found farther from the beach, within the fixed dune system (Fig. 1). By contrast, *P. schizantha* is restricted to interdune slacks within active coastal dune fields, where water accumulation—driven by precipitation and shallow groundwater—fosters diverse plant assemblages and creates microhabitats for local biota (Celsi and Giussani 2020).

To date, the delimitation of *Poa* entities has relied largely on a traditional morphological species concept grounded in phenetic similarity (Sokal and Crovello 1970, Giussani 2000). However, it has been difficult to identify a consistent set of diagnostic characters, as intermediate forms are commonly encountered (Giussani 2000, Giussani *et al.* 2000, Fernández Pepi *et al.* 2008, Giussani *et al.* 2012, Scrivanti *et al.* 2014, Finot *et al.* 2022b). Consequently, distinctiveness among species often extends beyond morphology to include ecological, phenotypic, physiological, or genetic differentiation, making the establishment of an operational working definition challenging (Walker *et al.* 2024).

**Figure 2:**
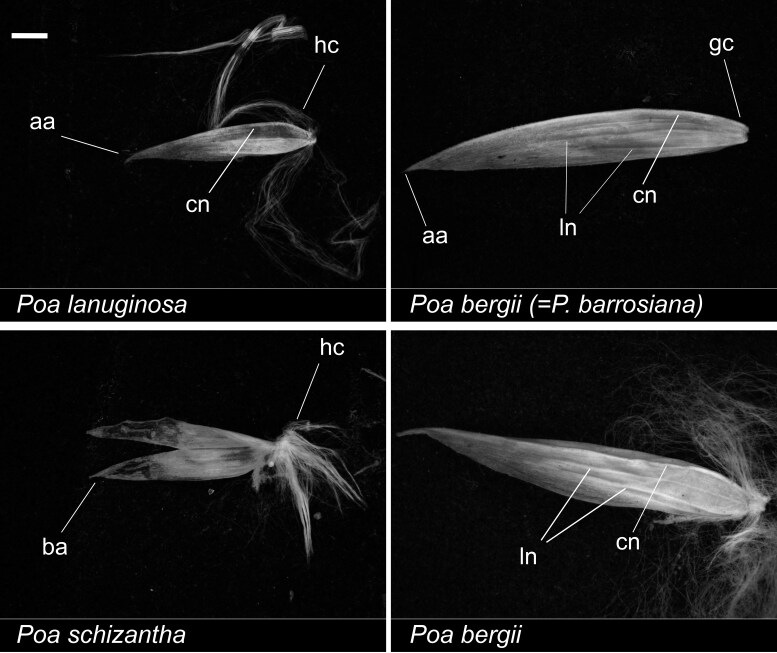
Lemma morphology in the studied taxa. The images illustrate differences among species in lemma size and shape, and qualitative traits. Apex morphology: aa, acute; ba, deeply bilobed. Callus pubescence in pistillate florets: gc, glabrous; hc, hairy or pubescent. Lemma nerves: cn, central nerve; ln, lateral nerves. Scale bar = 1 mm.

These three dune *Poa* species exhibit ecologically specialized traits, including clonal growth and long rhizomes that penetrate deeply into the sand, enabling persistence under sand movement and erosion (Herben and Klimešová 2020). Additionally, species identification is primarily based on quantitative morphological traits that display a continuous range of variation centred around the most frequent values (Giussani 2000, Giussani *et al.* 2012). *Poa bergii* differs from *P. lanuginosa* and *P. schizantha* by its more robust, taller plants, spikelets with larger anthoecia, and lemma with 7–9 nerves compared to the five nerves observed in the latter species (Fig. 2; Table 1). *Poa schizantha* stands out among *Poa* spp. due to its bilobate lemma, an interrupted panicle with long internodes, and a distinctive leaf blade anatomy lacking differentiated bulliform cells (Parodi 1940, Torres 1970). However, individuals may exhibit intermediate combinations of these diagnostic characters (Giussani 2000, Giussani *et al.* 2016, Sabena 2019), rendering species identification ambiguous and raising the question of whether such morphological intermediacy reflects hybridization, phenotypic plasticity, or early stages of divergence.

**Table 1:** Morphological differentiation among the three dioecious *Poa* species inhabiting coastal dune systems.

Morphological characters	*P. bergii—*BE	*P. lanuginosa—*LA	*P. schizantha—*SCH
Vegetative			
Leaf length, cm	35–60(–80)	15–60(–90)	20–35
Ligule length, mm	10–20(–25)	(2–)5–10(–17)	5–12
Culm length, cm	40–70 (–100)	20–60(–75)	(15–)30–50
Panicle length, cm.	10–20(–30)	5–15(–20)	15–25
Panicle shape	Contracted, dense, branches adpressed to the rachis	Contracted, dense, branches adpressed to the rachis	Contracted and interrupted (long internodes)
Blade width, mm	2–5	1.5–3	1–2
Bulliform cells in transverse view	Differentiated	Differentiated	Not differentiated
Reproductive			
Spikelets length, mm	(6–)8–12	(5–)6–8	8–10(–12)
Number of flowers per spikelet	5–11	3–7(–9)	4–8(–15)
Glume I, length, mm^a^			
Pistillate	7–10	4–6	(4–)5–6
Staminate	4–7	3–5	3.5–4
Number of nerves in glume I	3–5(–7)	3	1–3
Lower lemma length, mm^a^			
* Pistillate*	7–9.5	4.8–7	5–7
* Staminate*	5–7	4–5.5	4.5–5
Lemma apex	Acute, entire	Acute, entire	Deeply 2-lobed
Number of nerves in lower lemma	(5–)7–9	5	5
Hairs on floret callus^a^			
* Pistillate*	Woolly and long, more than half the floret, or glabrous (=*P. barrosiana*)	Woolly and long, more than half the floret	Rigid, straight, and long, more than half the floret
* Staminate*	Usually glabrous	Usually glabrous	Usually glabrous
Hairs on lemma nerves^a^			
* Pistillate*	Hairs more than 0.5 mm long or absent (= *P. barrosiana*)	Hairs less or more than 0.5 mm	Absent
* Staminate*	Absent	Usually absent	Absent

Main diagnostic characters follow the taxonomic treatments of Torres (1970), Giussani (2000), and Giussani *et al.* (2012). Characters marked with a designator (^a^) are sexually dimorphic and are presented separately for pistillate and staminate individuals.

Whole-genome duplication has played a major role in the evolution of many angiosperm families and is frequently associated with enhanced diversification through neofunctionalization, greater phenotypic variation, and ecological innovation (Van de Peer *et al.* 2009, Soltis *et al.* 2015, Soltis and Soltis 2016). In *Poa*, ∼91% of species are polyploids (Soreng *et al.* 2010), a condition widely reported across the genus (Stebbins 1950, Joshi *et al.* 2017). Species in section *Dioicopoa* are no exception: polyploidy is the predominant condition in this lineage (e.g. Saura 1948, Bowden and Senn 1962, Hunziker 1978, Guillin *et al.* 1995), likely contributing to their ecological success and genetic variability. Notably, no diploid species are currently known within this section (Soreng *et al.* 2010, Joshi *et al.* 2017), a pattern that may have facilitated ecological differentiation and reticulate evolution.

A phylogenetic approach based on plastid and nuclear molecular markers enabled the inference of independent lineages of dioecious *Poa* species (Giussani *et al.* 2016). The dune-inhabiting dioecious species of sect. *Madropoa*—*Poa douglasii* Nees and *Poa macrantha* Vasey, distributed along the Pacific coast of North America (Soreng 2007)—are distantly related to the Pampean dune species (*P. bergii*, *P. lanuginosa*, and *P. schizantha*) and to *Poa cumingii* Trin. from the South Pacific Chilean coast (Giussani *et al.* 2016). These results support independent evolutionary origins of dioecious dune-adapted *Poa* lineages in the Americas. Multilocus analyses including the nuclear ribosomal internal transcribed spacer (ITS) and external transcribed spacer (ETS) markers and plastid *trn*T-L and *trn*L-F regions provided limited resolution among dune species likely reflecting their recent divergence (Giussani *et al.* 2016). Therefore, traditional molecular markers may lack sufficient resolution to distinguish closely related and potentially reticulated lineages. Genome-wide approaches provide an effective framework for testing lineage boundaries (Bock *et al.* 2023), detecting hybridization and ongoing gene flow (Stull *et al.* 2023), characterize domesticated and wild relatives (Sassone *et al.* 2022), and assessing whether the currently recognized taxa constitute independently evolving lineages (Wu and Mao 2025).

The limited molecular differentiation but pronounced morphological divergence of *P. schizantha* suggests a notable case of recent speciation, involving greater morphological and ecological specialization than that observed in the other endemic species, *P. bergii*. Because the coastal dunes represent a geologically young landscape, divergence among these taxa is expected to be recent, with partial genomic admixture and weak lineage boundaries predicted among some species. In this study, we conducted a population-level phylogenomic analysis to assess genetic diversity and lineage relationships among *Poa* species inhabiting the southernmost Atlantic coastal dune ecosystems. Specifically, we tested whether the three dune species represent independently evolving lineages, quantified their genomic differentiation, and assessed the extent of hybridization within coastal dunes.

## Materials and methods

### Geographical sampling location

In Buenos Aires province, the sampling was conducted along 650 km in the maritime coastline, extending from Punta Rasa at the northern limit (36° 17ʹ 30ʹʹ S, 56° 46ʹ 42ʹʹ W) to ‘Villa Balnearia 7 de Marzo’ at the southern end (41° 1ʹ 23ʹʹ S, 62° 45ʹ 49ʹʹ W). Buenos Aires coastline is characterized by two dune barriers, the Northeastern and Southern dune barriers (NDB and SDB, respectively) that originated during the Middle Holocene as a consequence of sea-level fluctuations (Isla *et al.* 2001, Ponce *et al.* 2011). These dune barriers are separated by the southeastern tip of the Tandilia Range, which reaches the coastline in Mar del Plata. The Buenos Aires NDB extends from Punta Rasa to Mar del Plata; its geomorphological features and native dune vegetation have been comprehensively described by Marcomini (2003) and Marcomini *et al.* (2017). The SDB occurs between Mar del Plata and Bahía Blanca, where dunes are located on top of ancient cliffs (Bértola and Cortizo 2005, Isla *et al.* 2018, Austrich *et al.* 2021, Isla and Cortizo 2023). The plant communities of the Pampean coastal dunes have been extensively documented by Celsi and Monserrat (2006, 2008) and Celsi (2016). Dunes continue south on the coast of Río Negro province (Carbone *et al.* 2007), and from there on, dunes are reduced to patches or extended beaches in the Chubut province (del Valle *et al.* 2008, Favier Dubois and Borella 2011); this type of coast alternates between cliffs and bays (Isla *et al.* 2023).

### Species sampling

Specimens of *P. bergii*, *P. lanuginosa*, and *P. schizantha* were sampled across contrasting microhabitats described for each species (in the foredune, active dune, interdunal slacks, and inland) at multiple localities in the Argentine provinces of Buenos Aires, Chubut, and Río Negro. Voucher numbers and sampling localities are provided in Supplementary Table S1 and in Fig. 9. Plants were collected every time a population of any of the three species was found. When possible, staminate and pistillate specimens of the species were collected at the same site, ensuring a minimum distance of 10 m between individuals to guarantee appropriate spatial separation. Plants were collected during the blooming season, from spring to early summer. Species identification followed Torres (1970), Giussani (2000), and Giussani *et al.* (2012); a list of characters that distinguish the species is presented in Table 1. When character states were ambiguous, specimens (individuals/plants) were designated as ‘intermediate’ rendering species-level identification uncertain. Voucher specimens from each collection site were stored at the herbarium of the Instituto de Botánica Darwinion (SI) (Index Herbariorum, updated continuously), and leaves and inflorescences of every individual were preserved in silica gel (Supplementary Table S1).

### Genotyping-by-sequencing

Genomic DNA was extracted from silica gel–stored leaf tissue following a modified cetyltrimethylammonium bromide protocol as described in Giussani *et al.* (2016). For library preparation, the DNA quality and concentration were analysed with a Qubit analyser (Life Technologies Corporation, Carlsbad, CA, USA). Genotyping-by-sequencing (GBS; Elshire *et al.* 2011) followed a protocol based on two restriction enzymes, PstI-HF (CTGCAG, NEB Inc., Ipswich, UK) and MspI (CCGG, NEB Inc.), to digest 20 ng genomic DNA as described in Sassone *et al.* (2021). Barcoding and single-end sequencing were conducted on the Illumina NovaSeq 6000 according to Wendler *et al.* (2014). The GBS library construction and sequencing were carried out at the Leibniz Institute of Plant Genetics and Crop Plant Research in Germany. Quality assessment of 93 raw sequences was carried out using FastQC (Andrews 2010).

GBS loci were *de novo* assembled using the Ipyrad version 0.9.93 pipeline (Eaton and Overcast 2020). Parameters were configured based on recommendations from the Ipyrad documentation and by testing various settings as outlined in Eaton *et al.* (2017) and Gargiulo *et al.* (2021). All parameter files used are available at (10.5281/zenodo.20446293). In summary, assemblies were created using clustering thresholds of *c* = 0.95 and different numbers of minimum samples per locus (Table 2). Statistical base calling was conducted with a maximum of five *N*s in consensus sequences.

**Table 2:** Summary statistics of the GBS datasets.

Dataset	No. samples	No. loci	Concatenated length (bp)	% Missing data	No. SNPs	Min_samples/locus
1—Ingroup + OUT	90 + 3	198 767	20 429 599	74.33	1 048 880	4
2—Ingroup	90	37 325	3 907 344	25.77	350 126	45
3—Ingroup^a^	79	26 698	2 834 194	18.89	259 209	50
4—*P. bergii*	41	25 235	2 673 725	13.68	151 163	30
5—*P. schizantha*	19	50 921	5 327 516	20.26	100 573	10
6—*P. lanuginosa*	19	28 203	2 927 690	24.05	179 041	10

For each dataset, the table indicates the number of samples included, total number of loci recovered, concatenated alignment length (bp), percentage of missing data, total number of SNPs retained, and the minimum number of samples required per locus.

^a^Dataset 3 excludes intermediate individuals. Species-specific datasets correspond to independent assemblies for *P. bergii*, *P. schizantha*, and *P. lanuginosa*. Ingroup includes specimens of the three species and intermediates. Outgroup (OUT): 2 specimens of *P. lanigera* + *P. reitzii* (*Dioicopoa*).

### Phylogenomic inference

The maximum-likelihood (ML) analysis was conducted on Dataset 1 (Table 2), comprising 90 ingroup *Poa* samples and three outgroup accessions (two from *Poa lanigera* Nees and *Poa reitzii* Swallen), as implemented in FastTree v2.1.11 (Price *et al.* 2010) under the General Time Reversible (GTR) substitution model (‘-nt -gtr’ parameter). Branch support was assessed with 1000 resamples of the Shimodaira–Hasegawa local support.

### Phylogenetic network analysis using a distance approach

We generated a Neighbor-Net phylogenetic network given a distance matrix based on the neighbour-joining algorithm of Saitou and Nei as an alternative representation to evolution (Bryant and Moulton 2004). The network was generated based on DNA sequence data and derived from uncorrected P-distances using split-stree5 version 5.1.4-beta (Huson and Bryant 2006). Distances were calculated as the proportion of nucleotide sites at which two sequences differ, without applying a substitution model (Nei and Kumar 2000). A single network can represent several trees simultaneously, where there is conflict among different solutions, indicating possible hybridization or reticulation events (Bryant *et al.* 2007).

### Tests for introgression

**Figure 3:**
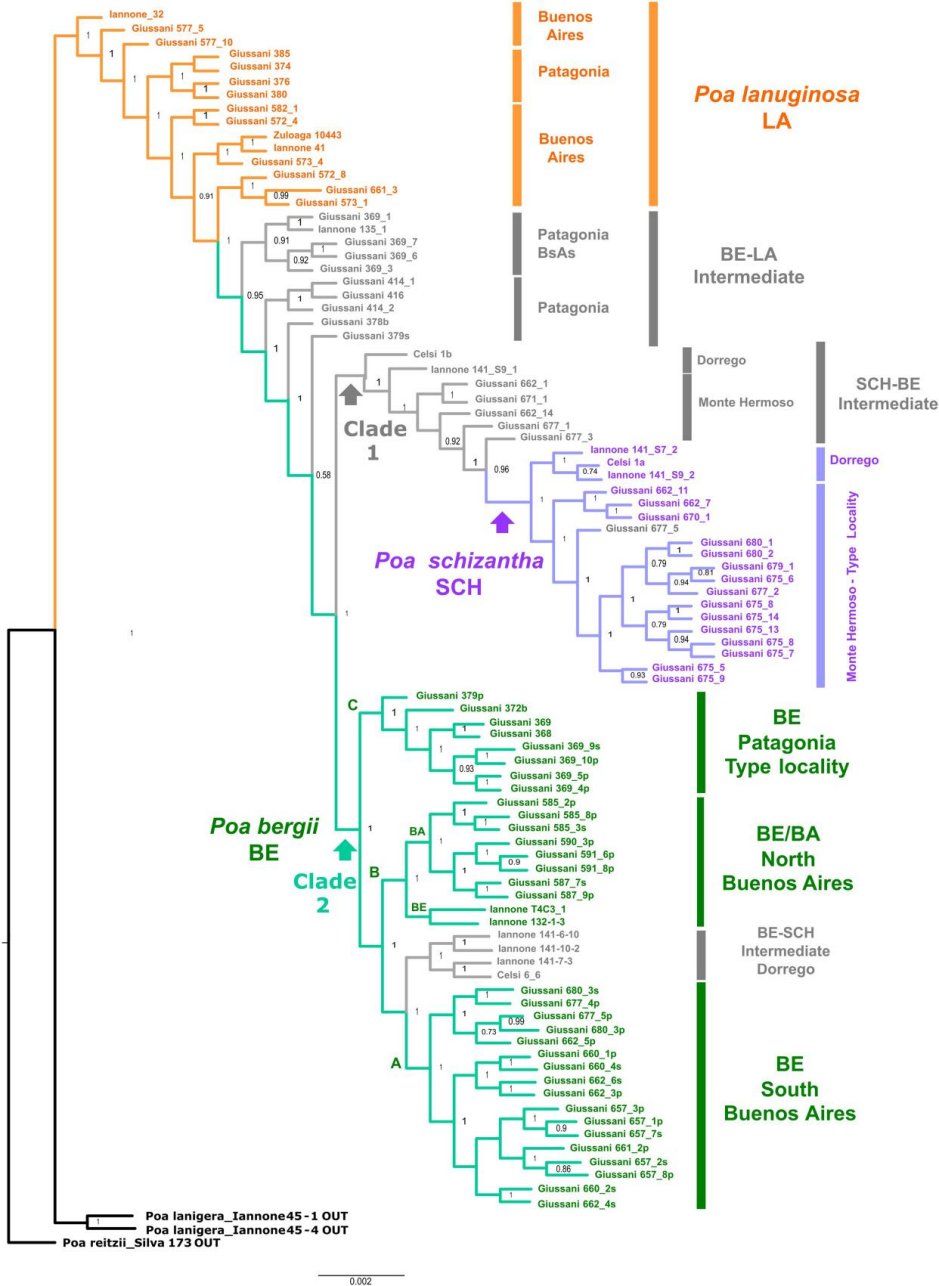
Maximum-likelihood phylogram inferred from GBS data for 93 samples, including 90 ingroup and 3 outgroup accessions (dataset 1; Table 2). Branch support values based on the Shimodaira–Hasegawa test are shown above branches when ≥0.9. BE-LA indicates specimens with intermediate morphology between *P. bergii* (BE) and *P. lanuginosa* (LA). SCH-BE and BE-SCH indicate specimens with intermediate morphology between *P. schizantha* (SCH) and *P. bergii* (BE), corresponding to Clades 1 and 2, respectively. BA indicates specimens of *P. bergii* (=*P. barrosiana*). Terminal labels include voucher information (collector name and number), and bars indicate geographic origin; complete details are provided in Supplementary Table S1.

To further investigate genetic connectivity among populations, we calculated Patterson's *D* statistics and the f4-ratio (ABBA-BABA) using Dsuite software (Malinsky *et al.* 2021). The Variant Call Format (VCF) file was filtered to retain only biallelic loci with <20% missing data and a minimum depth of at least 1000 across all samples resulting in a dataset of 139 262 SNPs. The input tree consisted of a collapsed phylogenetic tree summarizing these clusters: [(((((SCH-BE,SCH), (BE-SCH,BE)), BE-LA), LA), Outgroup)] (acronyms as in Fig. 3 and Supplementary Table S1). A total of 20 trios of taxa were analysed. In the ABBA-BABA framework, derived alleles are shared between P2 and P3 (ABBA) or P1 and P3 (BABA). Under a strictly bifurcating model, both patterns are expected at equal frequencies. Deviations from this expectation, reflected in significant *D* values, indicate introgression between P3 and either P1 or P2 (Malinsky *et al.* 2021). Statistical significance was assessed using a block-jackknife procedure implemented in Dsuite, and *Z*-scores were used to evaluate significance (|*Z*| > 3). We also calculated the f-branch (Malinsky *et al.* 2018), which allows the assignment of gene flow to specific, potentially internal, branches within a phylogeny.

### Population structure analyses

Population structure was assessed using both model-based and multivariate approaches. Analyses were conducted on the ingroup dataset (Dataset 2, Table 2) and on subsampled datasets (Datasets 4–6, Table 2).

The Bayesian clustering analysis was performed in STRUCTURE v2.2.4 (Pritchard *et al.* 2000), implemented through the IPYRAD Application Programming Interface (API), to infer the number of genetic clusters (K) and admixture proportions. Single Nucleotide Polymorphism (SNP) datasets were filtered to retain unlinked loci, in accordance with model assumptions of Hardy–Weinberg and linkage equilibrium. Each run consisted of 200 000 burn-in iterations followed by 5 000 000 Markov chain Monte Carlo (MCMC) iterations, with 10 independent replicates for each *K* value (*K* = 1–10).

Discriminant Analysis of Principal Components (DAPC), implemented in the adegenet package in R (Jombart 2008, Jombart *et al.* 2010), was applied to Datasets 4–6 (Table 2). Given the polyploid nature of the taxa, DAPC was used because it does not rely on Hardy–Weinberg or linkage equilibrium assumptions and incorporates a large number of loci, providing a robust description of genetic structure. The method uses principal component analysis (PCA) to summarize genetic variation, followed by discriminant analysis to maximize among-group variation. The number of retained principal components was determined using a cross-validation procedure with 1000 replicates.

### Measures of genetic diversity

Observed heterozygosity (Ho), expected heterozygosity (He), total heterozygosity (Ht), within-population inbreeding coefficient (FIS), and fixation index (FST) statistics were calculated using the R package ‘hierfstat’ (Goudet 2005). File conversions were performed using ‘dartR’ (Unmack *et al.* 2018). Datasets used to describe and compare overall, and population genetic diversity are indicated as 4, 5 and 6 (Table 2). Pairwise population FST values were estimated using the function stamppFst from the R package ‘StAMPP’ 1.6.2 (Pembleton *et al.* 2013), with 95% confidence interval estimated on 1000 bootstraps following Weir and Cockerham (1984). Only biallelic loci were retained for these analyses.

## Results

### Phylogenomic analyses

A ML phylogenetic reconstruction reveals *P. lanuginosa* (LA) ancestral among the coastal dune species, following a sequential evolutionary branching pattern (Fig. 3). This pattern is followed by specimens from the Patagonian coast, which prevent unambiguous assignment to either *P. bergii* or *P. lanuginosa* (e.g. intermediate BE-LA; Fig. 3; Supplementary Table S1). Then, two distinct clades diverged, each including two endemic dune species: *P. schizantha* (Clade 1) and *P. bergii* (Clade 2), together with morphological intermediate specimens.

Clade 1 includes intermediate specimens between *P. schizantha* and *P. bergii* (SCH-BE, specimens from Dorrego and Monte Hermoso), which appear as sisters to a well-supported clade with all unambiguously identified specimens of *P. schizantha* (sh = 1). Within the *P. schizantha* clade (SCH), two well-supported subclades are recovered, each corresponding to specimens from Dorrego and Monte Hermoso, respectively.

Clade 2 comprises a lineage containing *P. bergii* specimens (BE, sh = 1) and three subclades structured geographically (Fig. 3). The A subclade includes specimens from the Monte Hermoso locality and a sister lineage consisting of individuals showing intermediate morphology with *P. schizantha* (BE-SCH, specimens from Dorrego). The B subclade comprises specimens from the region between Miramar and Mar del Plata, which can be assigned to the synonymized species *Poa barrosiana* Parodi. This clade is sister to a minor group formed by two individuals collected from localities north and south of Mar del Plata (Faro Querandí and Claromecó, respectively) and identified as *P. bergii*. Based on morphological traits, *P. barrosiana* and *P. bergii* are characterized by large anthoecia and lemmas with 7–9 nerves; however, *P. barrosiana* is consistently distinguished by having pistillate glabrous anthoecia (Fig. 2). The third group, the C subclade, clusters specimens of *P. bergii* from the mouth of the Negro River (the species’ type locality)—as well as from neighbouring coastal areas.

**Figure 4:**
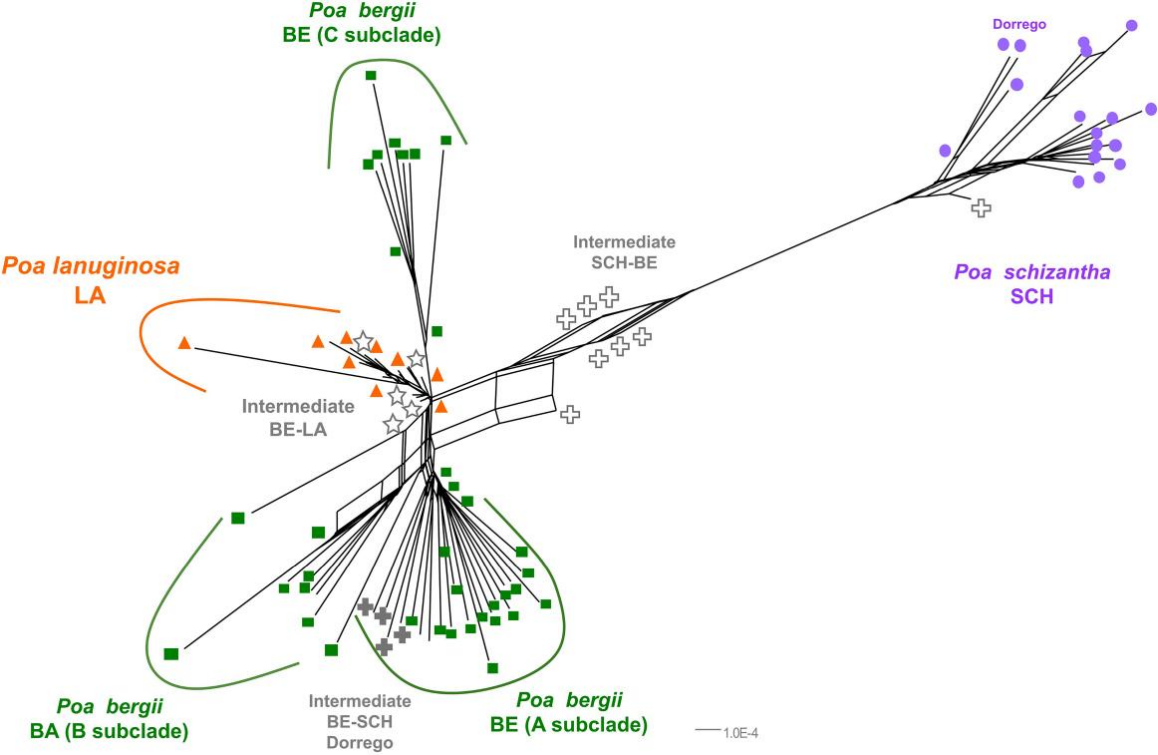
Neighbor-Net phylogenetic network inferred from GBS data (Dataset 2; Table 2), showing the major species groups recovered in Fig. 3 and reticulate relationships among taxa. Symbols and colours represent species as follows: triangles, *P. lanuginosa*; squares, *P. bergii*; circles, *P. schizantha*. Intermediate specimens are indicated by hollow stars between *P. bergii* and *P. lanuginosa* (BE-LA), hollow crosses between *P. schizantha* and *P. bergii* (Clade 1 in Fig. 3), and solid crosses between *P. bergii* and *P. schizantha* (Clade 2 in Fig. 3). Edge lengths are proportional to uncorrected p-distances.

The Neighbor-Net phylogenetic network generated from the GBS dataset 2 (Table 2) recovered a reticulate pattern (Fig. 4) interconnecting clades recognized in the phylogenetic tree (Fig. 3). The analysis shows a clade comprising all studied specimens of *P. schizantha*, including one individual with intermediate morphology at the base of the group. This clade appeared distantly related to the other two species, *P. bergii* and *P. lanuginosa*. Subclades A–C of *P. bergii,* as shown in Fig. 3, are also recovered as separate lineages. A reticulate pattern at the base of the clades is associated with intermediate specimens between *P. bergii* and *P. schizantha* (SCH-BE), as well as between *P. bergii* and *P. lanuginosa* (BE-LA).

### Introgression tests

Although the ML phylogeny recovered a hierarchical branching pattern, the Neighbor-Net analysis revealed reticulate connections among the same major groups, consistent with recent divergence. Based on previous genomic results, intermediate specimens were hypothesized to represent hybrids or introgressed individuals among the main species. To assess introgression, we performed ABBA-BABA tests within a phylogenetic framework derived from the major clades identified in Fig. 3 (collapsed phylogenetic tree in Newick format as in Materials and Methods).

The results revealed widespread and significant gene flow across the phylogeny (Supplementary Table S2). Introgression was consistently detected between the two main internal clades (Clade 1: SCH-BE and SCH and Clade 2: BE-SCH and BE), as well as between these clades and the more external groups including BE-LA (intermediate specimens between *P. bergii* and *P. lanuginosa*) and LA (*P. lanuginosa*). Most tested trios showed highly significant *D*-statistics (*Z*-scores > 3), indicating that gene flow is frequent among lineages rather than restricted to isolated cases (Supplementary Table S2).

**Figure 5:**
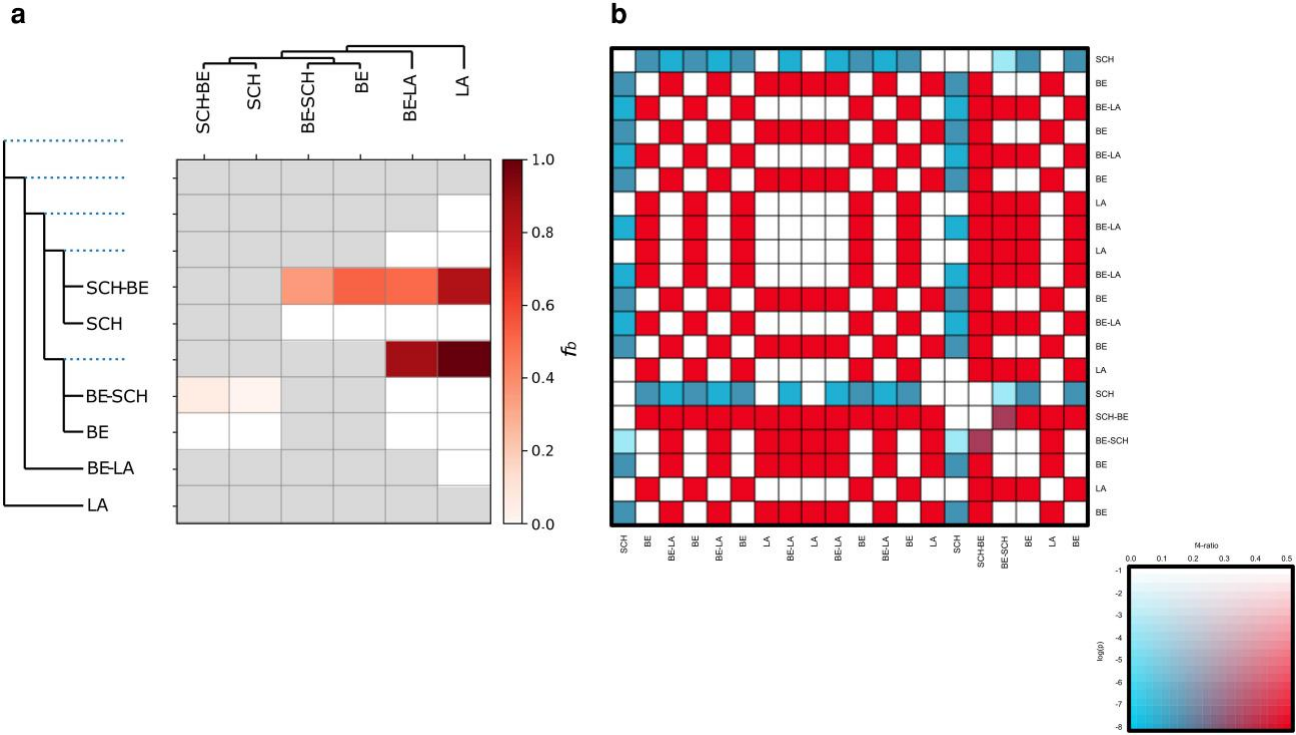
Introgression patterns inferred from ABBA-BABA analyses based on genome-wide SNP data. (a) Heatmap of significant introgression signals estimated with the *f_b_* statistic (f-branch) among major lineages and intermediate specimens. Warmer colours indicate stronger introgression signals. The SCH-BE lineage showed repeated introgression with *P. lanuginosa* (LA), *P. bergii* (BE), and intermediate BE-LA and BE-SCH specimens. Strong gene flow was also detected from the internal branch leading to the BE-SCH + *P. bergii* lineage into *P. lanuginosa* and BE-LA specimens. (b) Pairwise f4-ratio matrix showing the magnitude and significance of introgression among individuals and lineages.

The SCH-BE group (composed of intermediate specimens sister to *P. schizantha*) showed repeated signals of introgression with multiple taxa, suggesting an important role in the exchange of genetic material (Fig. 5a and b; Supplementary Table S3). Introgression signals were detected from SCH-BE specimens into *P. lanuginosa* (LA, *f* ≈ 0.84), intermediate BE-LA specimens (*f* ≈ 0.50), *P. bergii* (BE, *f* ≈ 0.52), and intermediate BE-SCH specimens (*f* ≈ 0.35). In contrast, *P. schizantha* (SCH) exhibited fewer and weaker introgression signals (Fig. 5a and b). Moreover, strong signals of gene flow were detected from the internal branch leading to the BE-SCH + *P. bergii* lineage into *P. lanuginosa* (LA, *f* ≈ 1) and intermediate BE-LA specimens (*f* ≈ 0.88). The observed f4-ratio supports both historical and recent introgression among these lineages. Overall, these results indicate a reticulate evolutionary history with multiple episodes of gene flow connecting both closely related and more divergent groups.

### Population structure analyses

Population structure analyses further supported a reticulate evolutionary pattern, revealing clear genetic differentiation among major groups, while also identifying individuals with admixed ancestry.

### Bayesian clustering analyses—STRUCTURE

**Figure 6:**
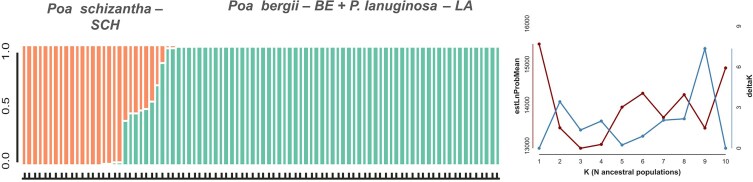
Population structure analyses based on 1086 unlinked SNPs. The Evanno plot from the STRUCTURE analysis indicated highest Δ*K* value *K* = 2, supporting the presence of two main genetic clusters and admixed specimens: *P. bergii* plus *P. lanuginosa* versus *P. schizantha*.

Population structure analyses based on 1086 unlinked SNPs revealed clear genetic differentiation among the studied taxa. Evanno's plot of the STRUCTURE analysis indicated that the highest Δ*K* was observed at *K* = 2, which is the most likely number of genetically distinct populations, largely corresponding to *P. bergii* plus *P. lanuginosa* versus *P. schizantha*. However, some individuals showed mixed ancestry, suggesting genetic connectivity between groups (Fig. 6).

Analyses were conducted separately for each grouping (Dataset: 4, 5, and 6, Table 2). Evidence of internal genetic structure was detected only within *P. schizantha* (Dataset 5, based on 5490 unlinked SNPs); Evanno’s plot indicated that the highest Δ*K* was observed at *K* = 2, supporting the presence of two main genetically distinct populations (Supplementary Fig. S1a). The STRUCTURE analysis of the *P. bergii* plus *P. lanuginosa* group showed weak genetic structure based on 2686 unlinked SNPs (Supplementary Fig. S1b).

### Discriminant analysis of principal components

**Figure 7:**
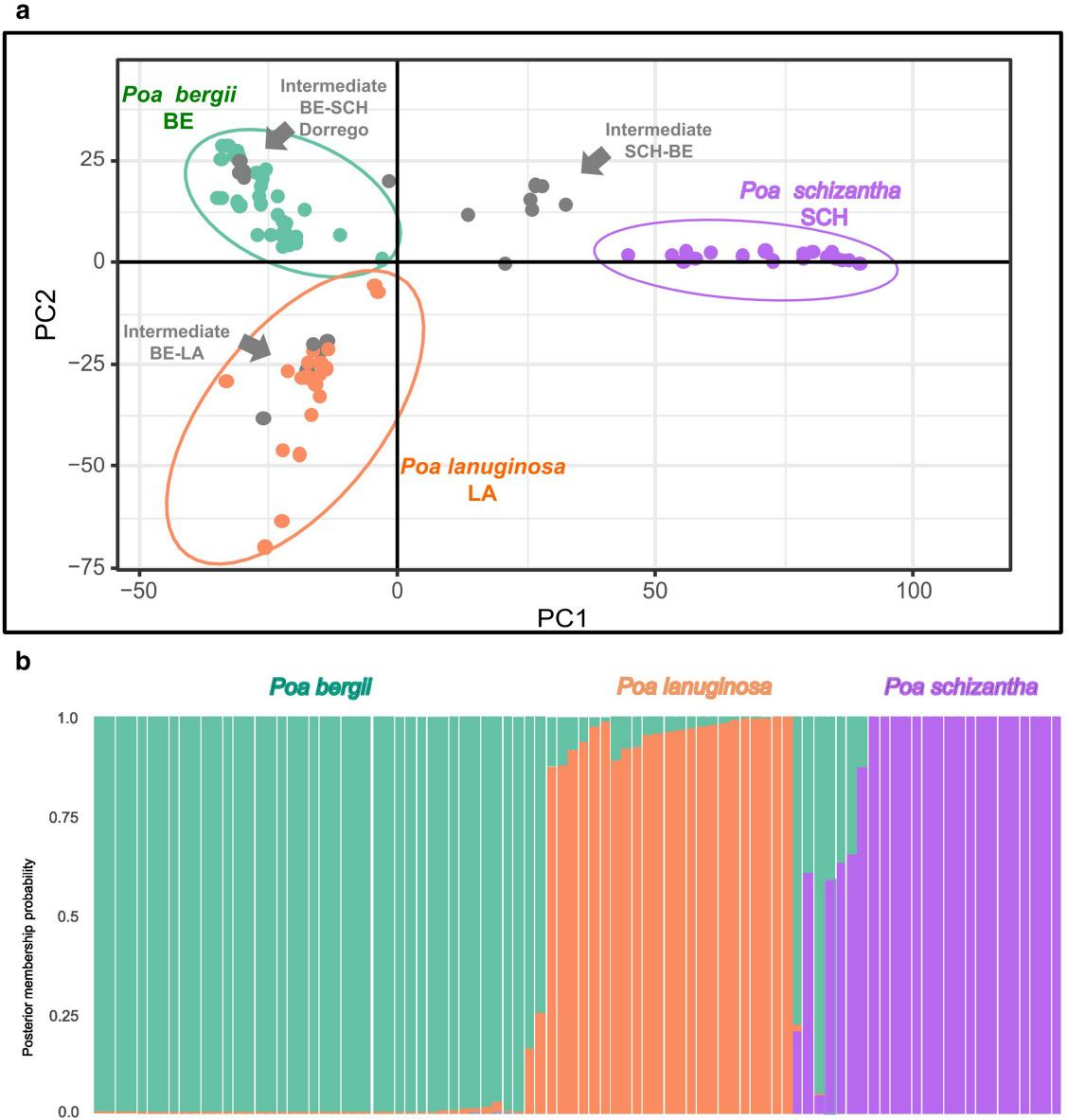
Population structure inferred from DAPC (Dataset 2, Table 2). (a) Scatterplot of the first two PCA showing the distribution of the 90 operational units. Individuals are coloured according to species assignment, whereas intermediate specimens are shown in grey. (b) Posterior membership probabilities estimated by DAPC. Each individual is represented by a vertical bar partitioned into coloured segments indicating the probability of assignment to each genetic cluster: *P. bergii*, green; *P. lanuginosa*, orange; *P. schizantha*: light violet.

The PCA based on 350 126 SNPs clearly differentiated the three dioecious *Poa* species along the first two components, which explained 30% of the observed variance (Fig. 7a). Moreover, the DAPCs distinguished the three species and revealed the presence of intermediate genotypes among them (Fig. 7b). While individuals of each species were generally well differentiated, evidence of admixture was detected, particularly between *P. bergii* and *P. lanuginosa*. A lower degree of admixture was also observed between *P. bergii* and *P. schizantha*, consistent with the phylogenetic and Neighbor-Net results (refer to Figs. 3 and 4).

### The endemic dune species

*Poa bergii* and *P. schizantha* are endemic to the Atlantic coastal dunes, whereas *P. lanuginosa*, a widespread species of sandy environments, represents their ancestral lineage (Fig. 9a). As indicated in previous genomic analyses (phylogenomic tree, Neighbor-Net, introgression tests, and population structure analyses), several specimens showed evidence of introgression among species, exhibiting intermediate genotypes. To better characterize the population structure of the endemic dune species, subsequent analyses focused on clearly assigned individuals, excluding admixed specimens with intermediate ancestry [Table 2, datasets 4 (*P. bergii*) and 5 (*P. schizantha*)]. Specifically, individuals showing admixture between *P. bergii* and *P. schizantha*, as well as between *P. bergii* and *P. lanuginosa*, were excluded to recover the underlying population structure of the endemic species.

### Population structure in *P. bergii*

**Figure 8:**
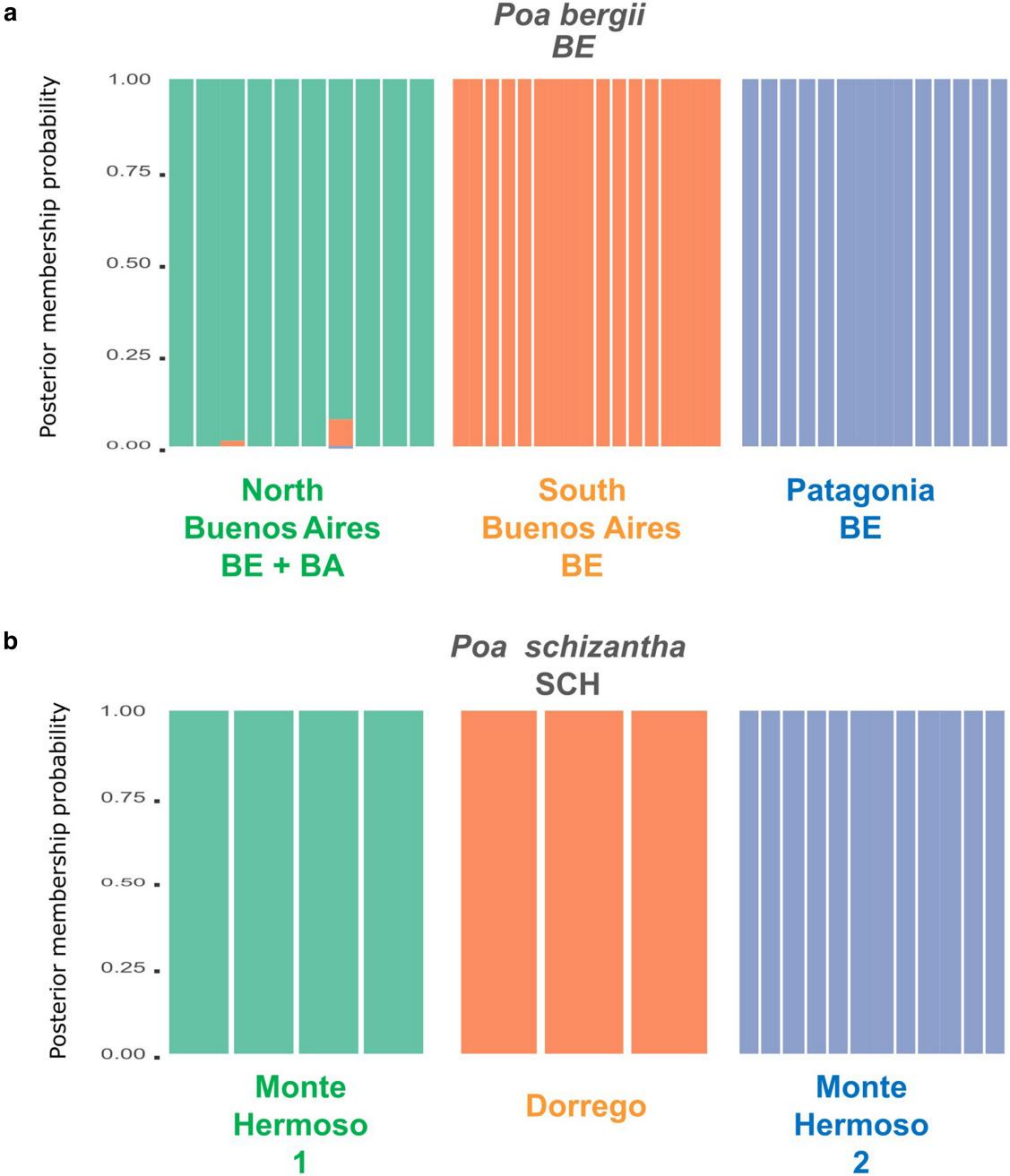
Population structure inferred from DAPC for each endemic species. Each individual is represented by a vertical bar partitioned into coloured segments indicating the probability of assignment to each genetic cluster. (a) Three genetic clusters or genotypes were identified within *P. bergii* across its distributional range, including northern Buenos Aires, southern Buenos Aires, and Patagonia. (b) Three genetic clusters were identified within *P. schizantha* populations from the Monte Hermoso (1 and 2) and Coronel Dorrego districts.

**Figure 9:**
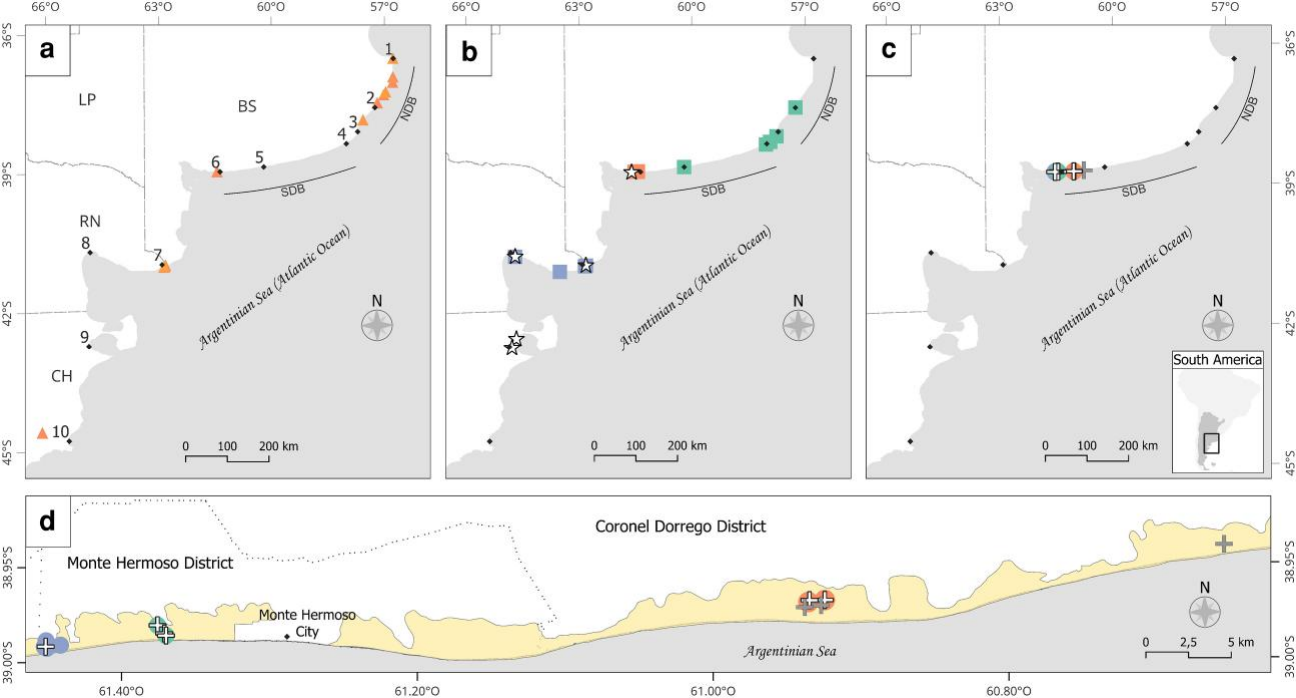
Geographic distribution of the three coastal dune species along the Atlantic coast of Argentina. (a) *P. lanuginosa*; (b) *P. bergii*; (c and d) *P. schizantha*. Coloured symbols in (b–d) indicate the genetic clusters identified by DAPC analyses, as described in the text and in Fig. 8 (a and b). Intermediate specimens are indicated by hollow stars (b): between *P. bergii* and *P. lanuginosa* (BE-LA); hollow crosses (c and d): between *P. schizantha* and *P. bergii* (Clade 1 in Fig. 3); and solid crosses (c and d) between *P. bergii* and *P. schizantha* (Clade 2 in Fig. 3). Solid diamonds are principal localities: 1: Punta Rasa; 2: Faro Querandí; 3: Mar del Plata; 4: Miramar; 5: Claromecó; 6: Monte Hermoso; 7: mouth of Negro River; 8: San Antonio Oeste; 9: Puerto Madryn; 10: Camarones. Province abbreviations: LP, La Pampa; BS, Buenos Aires; BS, Buenos Aires; RN, Río Negro; CH, Chubut.

Three distinct genotypes were identified within *P. bergii*, each associated with a specific geographic region (Figs. 8a and 9b). Individuals collected around Monte Hermoso exhibit a distinctive genotype, which is associated with the SDB near Pehuén Co and Monte Hermoso localities in Buenos Aires province, and corresponds to the A subclade (South-Buenos Aires BE) in Figs. 3 and 4. While specimens distributed between Mar del Plata and Miramar (North Buenos Aires BE + BA) occur in dunes spanning from the northern limit of the species’ distribution to the NDB of Buenos Aires province. This genotype corresponds to the B subclade in Figs. 3 and 4. The third genotype, represented by the southernmost populations in the coastal dunes from Patagonia up to Pehuén Co in Buenos Aires province (Patagonia BE), is associated with the C subclade (Patagonia-type locality BE) in Figs. 3 and 4.

### Population structure in *P. schizantha*

Three geographically structured populations were clearly identified within the narrow distribution of this species (Figs. 8b and 9c and d). A distinct genotype inhabits the dunes near Monte Hermoso 1 (green bars). A second genetically distinct group occurs at the western limit of the species’ range, far from Monte Hermoso 2 (blue bars). The third group comprises three individuals from the vicinity of Dorrego, located at the northeastern end of the species’ distribution (orange bars).

The two genotypes identified around Monte Hermoso are separated by ∼7 km along the coastal zone, whereas the Dorrego population is located ∼51 km far from the western limit of the species’ range, representing the greatest geographic distance among the sampled sites.

### Measures of genetic diversity

Summary statistics for the three *Poa* species—*P. bergii*, *P. lanuginosa*, and *P. schizantha*—were consistent with previous findings. The complete dataset was analysed excluding intermediate specimens (Dataset 3, Table 2). Overall observed heterozygosity (Ho = 0.0646) was slightly lower than expected heterozygosity (He = 0.0699), and total genetic diversity across species was Ht = 0.0868; although these estimates should be interpreted cautiously given the potential influence of ploidy variation in the group.

Genetic differentiation between species was estimated based on pairwise fixation indices (FST). FST values among the three species ranged from 0.084 to 0.3498. The highest levels of genetic differentiation were observed between *P. schizantha* and *P. bergii* (FST = 0.3498) and similarly between *P. schizantha* and *P. lanuginosa* (FST = 0.3387), indicating clear genetic divergence and likely reproductive isolation of *P. schizantha* from the other two species. In contrast, the lowest differentiation was found between *P. bergii* and *P. lanuginosa* (FST = 0.084).

Genetic diversity within species also differed among taxa. *Poa bergii* (Dataset 4), a species which has a broader geographical range than *P. schizantha*, showed lower observed than expected heterozygosity (Ho = 0.0952, He = 0.1093). Similarly, *P. schizantha* (Dataset 5) also showed lower heterozygosity than expected (Ho = 0.1715, He = 0.2044). Meanwhile, *P. schizantha* exhibited a slightly higher inbreeding coefficient (FIS = 0.1609) than *P. bergii* (FIS = 0.13) and the fixation index also exhibited higher (FST = 0.1537) than *P. bergii* (FST = 0.0505). Total genetic diversity was also higher in *P. schizantha* (Ht = 0.2416) than in *P. bergii* (Ht = 0.1115).

Genetic variation within *P. lanuginosa* was assessed only in coastal dunes and sandy coastal populations. In this species, observed and expected heterozygosity were very similar (Ho = 0.1468, He = 0.1439) (Dataset 6). The low FST value (0.005) indicates minimal differentiation among populations, with most variation distributed within populations. The slightly negative FIS (−0.0199) supports the absence of inbreeding.

These results highlight stronger genetic isolation and differentiation in *P. schizantha*, intermediate diversity and structure in *P. bergii*, and low structure with high connectivity in *P. lanuginosa* along the Atlantic coastal dunes in Argentina.

## Discussion

According to Giussani *et al.* (2016), phylogenetic analyses based on traditional molecular markers revealed that South American dioecious *Poa* species share a monophyletic origin, likely associated with a DNA duplication event derived from a polyploid (tetraploid) ancestor that diverged during the Late Pliocene to Early Pleistocene. This divergence led to the development of an outcrossing mating system, characterized by the segregation of male (staminate) and female (pistillate) reproductive organs into separate dimorphic individuals (Connor 1979, Anton and Connor 1995). These species were taxonomically classified within the *Poa* sect. *Dioicopoa*. Subsequently, they experienced evolutionary radiation during the Middle to Late Pleistocene. In this process, morphological distinctiveness evolved more rapidly than the fixation of molecular variation (Giussani *et al.* 2016). As a result, phylogenetic analyses based on multilocus sequence data revealed low resolution within the sect. *Dioicopoa*, highlighting the need for additional data or alternative approaches to evaluate whether the three dioecious *Poa* species inhabiting Atlantic coastal dunes share a recent divergence from a common ancestor. Such limited resolution is consistent with recent divergence, incomplete lineage sorting, and ongoing gene flow. We therefore interpret the studied taxa as representing a case of incipient speciation, likely driven by the dynamic and stressful habitat conditions of the coastal dunes. Furthermore, our analyses help resolve relationships among morphological intermediate groups and provide evidence of gene flow among sympatric or historically connected populations along the Atlantic coastal dunes.

Coastal dunes are recently formed landscapes that facilitate processes of colonization and plant successional dynamics. In Argentina, dune formation occurred through at least three major cycles from the Middle Holocene to the present (Isla and Espinosa 1995, Isla *et al.* 2001). The first cycle (ca. 6000–4000 years ago) occurred synchronously and at comparable latitudes with dune formation along the southern hemisphere coasts of Africa and Australia (Isla *et al.* 2001, Lewis *et al.* 2008, Cowling *et al.* 2023). Such phases of dune formation likely created new ecological opportunities, promoting lineage divergence. In this context, *P. lanuginosa*—a species adapted to sandy soils in Patagonia, ranging from the Andes to both the Atlantic and Pacific coasts (Giussani *et al.* 2012, Finot *et al.* 2022a, 2022b)—may represent the ancestral lineage from which the endemic dune species originated. Its presence in Atlantic coastal dunes likely reflects historical processes linked to sea-level regressions and transgressive pulses during the postglacial Holocene (Isla *et al.* 2001, Lima and Parise 2020).

As a result of the formation of these environments, speciation in dune plants can be interpreted within an evolutionary framework. The recovered topology is consistent with *P. lanuginosa* representing an ancestral, paraphyletic lineage from which recently differentiated dune taxa emerged (Fig. 3). In this context, paraphyly should not be viewed as a taxonomic impediment to species recognition, but rather as an expected outcome of lineage divergence, given that speciation may proceed through paraphyletic phases (Rieseberg and Brouillet 1994). During this process, traits such as morphological distinctiveness, reproductive isolation, monophyly, and ecological specialization may arise during species evolution (de Queiroz 2007). Accordingly, recognizing *P. lanuginosa* as a paraphyletic species preserves its current circumscription and is consistent with the ‘paraspecies’ concept, in which a widespread ancestral species gives rise to one or more derived species without itself becoming extinct (Ackery and Vane-Wright 1984, Crisp and Chandler 1996, Albert and Reis 2011). Recognizing *P. bergii* and *P. schizantha* as recent species derived from *P. lanuginosa* is consistent with a peripheral or ecological speciation associated with the emergence of coastal dune ecosystems. As noted by Levin (2004), it is essential to consider the geographical and ecological context in which speciation occurs; he proposed a two-stage process of ecological speciation involving the colonization of a new habitat followed by genetic and phenotypic refinement that promotes adaptation to local conditions and ultimately leads to species formation. Microhabitat heterogeneity within dune fields likely promoted the selection of novel genotypes and increased endemism. Both dune-endemic lineages, *P. bergii* and *P. schizantha*, exhibit increasing morphological and molecular differentiation, while still retaining signals consistent with recent or ongoing gene flow across foredunes, interdune slacks, and active dune fields. This pattern is consistent with global trends in semi-arid coastal dune systems, which typically sustain low overall species richness but relatively high levels of endemism (Hesp 1991).

At the population level, contrasting patterns of genetic structure become evident among species. Coastal populations of *P. lanuginosa* display high levels of gene flow and minimal genetic differentiation, likely facilitated by effective dispersal mechanisms. As in most grasses, *P. bergii*, *P. schizantha*, and *P. lanuginosa* are likely wind-pollinated, while seed dispersal may occur through wind, substrate movement, and local water transport across dune habitats. This pattern is consistent with evidence from coastal species like *Calibrachoa heterophylla* (Sendtn.) Wijsman, where wind corridors drive genetic admixture and connectivity among populations (Silva-Arias *et al.* 2021). Connectivity along inland and coastal dunes, together with the phylogenomic position of *P. lanuginosa* and signals of introgression, support this species as an ancestral lineage of both derived dune-endemic lineages.

*Poa bergii* and *P. schizantha* exhibit stronger population genetic structure than *P. lanuginosa*, as evidenced by the presence of distinct genetic groups distributed across their respective geographical ranges (Fig. 9). In contrast, *P. bergii* occupies a broader range of coastal dune habitats, usually found within active dunes; it shows geographically structured populations, consistent with fragmentation, regional isolation, and local differentiation along the dune corridor. In *P. bergii*, population structure analyses identified geographically differentiated genotypes (e.g. South-Buenos Aires BE, Patagonia-Type BE, or North-Buenos Aires BA) suggesting population fragmentation and hierarchical structure, possibly driven by historical isolation and local selective pressures. Notably, the North-Buenos Aires (BA) subclade (Figs. 3 and 4) corresponds to a distinct genetic group that includes individuals with glabrous pistillate lemmas (Fig. 2), a morphological trait associated with a previously synonymized *P. barrosiana* (Giussani 2000, Giussani *et al.* 2012), which may warrant taxonomic reassessment at the subspecific level.

Although genetic differentiation between *P. bergii* and *P. lanuginosa* is lower than that observed between *P. schizantha* and the other species, multiple lines of evidence support the recognition of both *P. bergii* and *P. schizantha* as independently evolving lineages that differ in the degree of divergence attained. *Poa bergii* represents a distinct but recently derived lineage within a continuum of divergence from the widespread ancestral species *P. lanuginosa*. By contrast, *P. schizantha* shows stronger genetic and morphological differentiation, consistent with a more advanced stage of speciation. Both Bayesian and discriminant approaches consistently identified a distinct genetic group in the Dorrego population, located at the northeastern edge of the species’ range (Fig. 9d). Within active coastal dunes, *P. schizantha* occurs in interdunal slacks, often subject to temporary flooding, suggesting ecological specialization to this microhabitat (Celsi and Giussani 2020, 2023). Although morphological variation has been documented in previous floristic treatments and the original species description (Parodi 1940, Torres 1970, Giussani 2000, Giussani *et al.* 2012, Celsi and Giussani 2020, 2023), no diagnostic morphological differences were detected among the three genetically distinct groups. Taken together, these findings support the interpretation of *P. schizantha* as a cryptic, locally adapted lineage undergoing early stages of divergence (Bickford *et al.* 2007, Peng *et al.* 2025).

Only from Monte Hermoso to Dorrego, all three species are present together in dune fields: *P. schizantha* is primarily associated with interdunal slacks within active dunes, suggesting adaptation to more specialized and heterogeneous microhabitats, while *P. bergii* and *P. lanuginosa* are found in foredunes and active dune fields surrounding interdunal slacks. Instead, *P. lanuginosa* extends inland into fixed dune grasslands. *Poa bergii* and *P. lanuginosa* are usually found together in active dune zones from the northern dune barrier in Buenos Aires to the coast of Chubut province (Fig. 9a and b).

### Hybridization in sympatric populations and morphological evidence of intermediacy

Despite this overall differentiation, several populations and individuals show evidence of admixture and morphological intermediacy, particularly in zones of sympatry. Specimens identified as putative hybrids exhibit intermediate morphological traits. For instance, some individuals display a bilobate lemma—a diagnostic feature of *P. schizantha*—but with shorter lobules than typically observed in that species. At the same time, they show a more robust habit and larger anthoecia with a seven-nerved lemma, resembling *P. bergii*, whereas their thinner leaves are more characteristic of *P. schizantha*. This combination of traits allows the recognition of morphologically intermediate individuals within the distributional range and habitat of *P. schizantha*. Consistent with the phylogenomic evidence, intermediates between *P. lanuginosa* and *P. bergii* show variation in reproductive traits such as anthoecium size, panicle and culm length, and number of lemma nerves.

The phylogenomic approach confirms genetic intermediacy and facilitates the detection of complex evolutionary patterns. One significant group showing introgression with almost all other groups is the intermediate SCH-BE, which is sister to *P. schizantha*. This group likely represents introgressed specimens between *P. bergii* and *P. schizantha* within their sympatric range, along the margins of the ecological niche of *P. schizantha*. In the surrounding areas of the interdunal slacks, *P. schizantha* co-occurs with both *P. bergii* and *P. lanuginosa,* resulting in introgressed individuals that mediate gene exchange between more recent and ancestral lineages.

Hybridization is frequent among closely related species generating reticulate evolutionary patterns that may obscure species boundaries (Soltis and Soltis 2009, Nosov *et al.* 2015). In *Dioicopoa*, this process is likely facilitated by the polyploid nature of species (Soltis *et al.* 2014), which contributed to diversification under environmental stress by enhancing adaptability while potentially promoting reproductive isolation (Alix *et al.* 2017, Islam *et al.* 2022, Tossi *et al.* 2022). Although direct cytogenetic information is not available for the specific accessions of *P. bergii* and *P. schizantha* analysed here, previous chromosome counts indicate that tetraploid and octoploid levels are widespread in *Dioicopoa*, suggesting a history of genome duplication within the group. Unresolved ploidy variation may influence the interpretation of our results; therefore, incorporating cytogenetic data will be essential for refining future evolutionary interpretations.

In the endemic dune species of *Poa*, ongoing gene flow and potential introgression indicate recent and possibly incomplete diversification, with hybridization still shaping their evolutionary relationships (Alix *et al.* 2017). Hybridization may reflect either incomplete reproductive isolation during recent divergence or secondary contact following the range expansion of *P. schizantha*, where it comes into contact and hybridizes with its closest relative. Despite this, lineages may coexist within the restricted distributional range of *P. schizantha*, likely because of niche differentiation among closely related species. Together with the presence of morphologically and genetically intermediate individuals, these patterns are consistent with early stages of lineage divergence. Rather than obscuring species boundaries, they highlight the dynamic nature of divergence in this system, where gene flow coexists with lineage differentiation.

### Genomics in conservation initiatives

This study highlights the genetic diversity of endemic species from the Atlantic coastal dunes, a highly threatened habitat due to increasing land-use pressures (Austrich *et al.* 2021). The application of next-generation sequencing technologies enables a detailed characterization of inter- and intraspecific genetic variation, thus supporting the integration of population genomics into early-stage conservation planning (Doyle *et al.* 2025).

In *P. bergii*, which may be considered vulnerable due to the ongoing fragmentation of its native habitat, this genomic approach is particularly valuable. Among the three genetically distinct groups identified, one exhibits unique morphological features and a highly restricted distribution. Accordingly, the reinstatement of *P. barrosiana* as a subspecies of *P. bergii* deserves particular attention and should be classified as Endangered (EN), given that it is confined to areas increasingly impacted by tourism and urban development (IUCN 2012).

The case of *P. schizantha* warrants special attention. The species was long considered extinct (Delucchi 2006) due to the absence of records for over 60 years, until it was rediscovered in 2002 and subsequently recorded (Celsi and Giussani 2023). Based on its extreme rarity, narrow distribution, and high habitat specificity, Celsi and Giussani (2020) emphasized the species’ conservation concern, particularly in light of ongoing threats such as the intense conversion of dune ecosystems into urban areas, vehicle traffic on dunes, and long-term afforestation practices along the Buenos Aires coast that have been carried out for nearly a century (Dadon and Matteucci 2009, Faggi and Dadon 2011, Austrich *et al.* 2021). Our results reveal high genetic variability structured into three geographically distinct groups within a narrow ∼51 km range. To ensure the conservation of this distinctive species—characterized by notable morphological and genotypic traits—we proposed that the species be classified as Critically Endangered (CR) under the criteria of the International Union for Conservation of Nature (IUCN) (Celsi and Giussani 2020).

To date, public funding has primarily supported advances in taxonomy, distribution, ecology, and genomics of these species. Moving forward, it is imperative to engage local communities and governmental authorities in conservation management and environmental education to safeguard not only these species but also the evolutionary differentiation processes within these increasingly threatened ecosystems.

## Supplementary Material

plag028_Supplementary_Data

## Data Availability

Raw sequence data are available at the European Nucleotide Archive (ENA) under project number PRJEB114505 and R code used for analyses is available at https://doi.org/10.5281/zenodo.20446293.

## References

[plag028-B1] Ackery PR, Vane-Wright RI. Milkweed Butterflies: Their Cladistics and Biology. Ithaca: Cornell University Press, 1984, 425.

[plag028-B2] Albert JS, Reis RE. Historical Biogeography of Neotropical Freshwater Fishes. University of California Press, 2011, 308.

[plag028-B3] Alix K, Gérard PR, Schwarzacher T et al Polyploidy and interspecific hybridization: partners for adaptation, speciation and evolution in plants. Ann Bot 2017;120:183–94. 10.1093/aob/mcx079. Erratum in: *Ann Bot*. 2017 Oct 17;**120**(4):619. doi:10.1093/aob/mcx096 28854567 PMC5737848

[plag028-B4] Andrews S. *FastQC: A Quality Control Tool for High Throughput Sequence Data*. 2010. http://www.bioinformatics.babraham.ac.uk/projects/fastqc (28 November 2025, date last accessed).

[plag028-B5] Anton AM, Connor HE. Floral biology and reproduction in *Poa* (Poeae: Gramineae). Aust J Bot 1995;43:577–99. 10.1071/BT9950577

[plag028-B6] Austrich A, Mapelli FJ, Mora MS et al Landscape change and associated increase in habitat fragmentation during the last 30 years in coastal sand dunes of Buenos Aires Province, Argentina. Estuaries Coast 2021;44:643–56. 10.1007/s12237-020-00798-x

[plag028-B7] Bértola GR, Cortizo LC. Transporte de arena en médanos litorales activos y colgados del sudeste de Buenos Aires. Revista de la Asociación Geológica Argentina 2005;60:174–84. https://revista.geologica.org.ar/raga/article/view/1023/1013

[plag028-B8] Bickford D, Lohman DJ, Sodhi NS et al Cryptic species as a window on diversity and conservation. Trends Ecol Evol 2007;22:148–55. 10.1016/j.tree.2006.11.004 17129636

[plag028-B9] Bock DG, Cai Z, Elphinstone C et al Genomics of plant speciation. Plant Commun 2023;4:100599. 10.1016/j.xplc.2023.100599 37050879 PMC10504567

[plag028-B10] Bowden WM, Senn HA. Chromosome numbers in 28 grass genera from South America. Can J Bot 1962;40:1115–24. 10.1139/b62-102

[plag028-B11] Bryant D, Moulton V. Neighbor-net: an agglomerative method for the construction of phylogenetic networks. Mol Biol Evol 2004;21:255–65. 10.1093/molbev/msh018 14660700

[plag028-B12] Bryant D, Moulton V, Spillner A. Consistency of the neighbor-net algorithm. Algorithms Mol Biol 2007;2:8. 10.1186/1748-7188-2-8 17597551 PMC1948893

[plag028-B13] Carbone ME, Perillo GME, Piccolo MC. Dinámica morfológica de los ambientes costeros de Bahía San Antonio Oeste, provincia de Río Negro. Geoacta 2007;32:83–91. https://ri.conicet.gov.ar/handle/11336/38332

[plag028-B14] Celsi CE . La vegetación de las dunas costeras pampeanas. In: Athor J, Celsi CE (eds.), La costa atlántica de Buenos Aires: naturaleza y patrimonio cultural. Buenos Aires, Argentina: Fundación de Historia Natural Félix de Azara, 2016, 116–38.

[plag028-B15] Celsi CE, Giussani LM. Geographical distribution and habitat characterization of *Poa schizantha* (Poaceae), a narrow endemic of the coastal sand dunes of the southern Pampas, Argentina. Bot J Linn Soc 2020;192:296–313. 10.1093/botlinnean/boz069

[plag028-B16] Celsi CE, Giussani LM. Pastito de los bajos (*Poa schizantha* Parodi). In: Dalia AV, Bauni V, Homberg M, Giacchino A (eds.), Dos décadas de trabajo con especies amenazadas de la Argentina. Buenos Aires: AZARA Fundación de Historia Natural Félix de Azara, 2023, 38–55. ISBN 978-987-8989-07-5.

[plag028-B17] Celsi CE, Monserrat AL. Valor y funcionalidad ecológicos de las dunas costeras de Coronel Dorrego, Buenos Aires. Bosque 2006;27:201–2. 10.4067/s0717-92002006000200011

[plag028-B18] Celsi CE, Monserrat AL. La vegetación dunícola en el frente costero de la Pampa Austral (Partido de Coronel Dorrego, Buenos Aires). Multequina 2008;17:73–92. https://www.scielo.org.ar/pdf/multeq/v17n2/v17n2a05.pdf

[plag028-B19] Connor HE . Breeding systems in the grasses: a survey. N Z J Bot 1979;17:547–74. 10.1080/0028825X.1979.10432571

[plag028-B20] Cowling RM, Cawthra H, Privett S et al The vegetation of Holocene coastal dunes of the Cape south coast, South Africa. PeerJ 2023;11:e16427. 10.7717/peerj.16427 38107568 PMC10722985

[plag028-B21] Crisp MD, Chandler G. Paraphyletic species. Telopea 1996;6:813–44. 10.7751/telopea19963037

[plag028-B22] Dadon JR, Matteucci SD. Coastal zone management in Buenos Aires, Argentina. Ocean Yearbook Online 2009;23:361–88. 10.1163/22116001-90000200

[plag028-B23] Delucchi G . Las especies vegetales amenazadas de la Provincia de Buenos Aires: Una actualización. APRONA Boletín Científico 2006;39:19–31.

[plag028-B24] del Valle HF, Rostagno CM, Coronato FR et al Sand dune activity in north-eastern Patagonia. J Arid Environ 2008;72:411–22. 10.1016/j.jaridenv.2007.07.011

[plag028-B25] De Queiroz K . Species concepts and species delimitation. Syst Biol 2007;56:879–86. 10.1080/10635150701701083 18027281

[plag028-B26] Doyle CAT, Cascini M, Yap JYS et al Conservation genomics within government led conservation planning: an Australian case study exploring cost and benefit for threatened flora. Ann Bot 2025;135:1229–42. 10.1093/aob/mcae222 39806772 PMC12259528

[plag028-B27] Eaton DAR, Overcast I. Ipyrad: interactive assembly and analysis of RADseq datasets. Bioinformatics 2020;36:2592–4. 10.1093/bioinformatics/btz966 31904816

[plag028-B28] Eaton DAR, Spriggs EL, Park B et al Misconceptions on missing data in RAD-seq phylogenetics with a deep-scale example from flowering plants. Syst Biol 2017;66:399–412. 10.1093/sysbio/syw092 27798402

[plag028-B29] Elshire RJ, Glaubitz JC, Sun Q et al A robust, simple genotyping-by-sequencing (GBS) approach for high diversity species. PLoS One 2011;6:e19379. 10.1371/journal.pone.0019379 21573248 PMC3087801

[plag028-B30] Faggi A, Dadon J. Temporal and spatial changes in plant dune diversity in urban resorts. J Coast Conserv 2011;15:585–94. 10.1007/s11852-011-0148-1

[plag028-B31] Favier Dubois CM, Borella F. Contrastes en la costa del golfo: una aproximación al estudio del uso humano del litoral rionegrino en el pasado. Capítulo 1. In: Cardillo M, Borella F (eds.), Arqueología de pescadores y marisqueadores en Nordpatagonia. Buenos Aires: Editorial Dunken, 2011, 13–42.

[plag028-B32] Fernández Pepi MG, Giussani LM, Morrone O. Morphological variability of *Poa resinulosa* species complex (Poaceae) and relationships with sect. Dioicopoa species. Darwiniana 2008;46:279–96. 10.14522/darwiniana.2014.462.74

[plag028-B33] Finot VL, Giussani LM, Soreng RJ. *Poa* L. In: Rodríguez R, Marticorena A (eds.), Flora de Chile 6(1) I-Z. Chile: Universidad de Concepción, 2022a, 821–915.

[plag028-B34] Finot VL, Soreng RJ, Giussani LM et al Taxonomic revision of the genus *Poa* L. (Poaceae: Pooideae: Poeae) in Chile. Gayana Botanica 2022b;79:159–253. 10.4067/S0717-66432022000200159

[plag028-B35] Gargiulo R, Kull T, Fay MF. Effective double-digest RAD sequencing and genotyping despite large genome size. Mol Ecol Resour 2021;21:1037–55. 10.1111/1755-0998.13314 33351289

[plag028-B36] Giussani LM . Phenetic similarity patterns of dioecious species of *Poa* from Argentina and neighboring countries. Ann Mo Bot Gard 2000;87:203–33. 10.2307/2666161

[plag028-B37] Giussani LM, Gillespie LJ, Scataglini MA et al Breeding system diversification and evolution in American *Poa* supersect. *Homalopoa* (Poaceae: Poeae: Poinae). Ann Bot 2016;118:281–303. 10.1093/aob/mcw108 27373539 PMC4970369

[plag028-B38] Giussani LM, Negritto MA, Romanutti A et al *Poa*. In: Zuloaga FO, Rúgolo de Agrasar ZE, Anton AM (eds.), Flora Argentina. Flora Vascular de la República Argentina, Monocotyledoneae. Poaceae. Pooideae, Vol. 3. Córdoba: Gráficamente Ediciones, 2012, 284–339.

[plag028-B39] Giussani LM, Nicora EG, Roig FA. *Poa durifolia* y su relación con el patrón fenético de *Poa* sección Dioicopoa (Poaceae). Darwiniana 2000;38:47–57. https://www.ojs.darwin.edu.ar/index.php/darwiniana/article/view/161

[plag028-B40] Goudet J . HIERFSTAT, a package for R to compute and test hierarchical F-statistics. Mol Ecol Notes 2005;5:184–6. 10.1111/j.1471-8286.2004.00828.x

[plag028-B41] Guillin EA, Giussani LM, Oliva G. Estudios morfológicos y citológicos en especies dioicas del género *Poa*. In: *XXVI Congreso Argentino de Genética y I Jornada Argentino–Chilena de Genética. XXVIII*. Río Negro, Argentina: Sociedad Argentina de Genética San Carlos de Bariloche, 1995.

[plag028-B42] Herben T, Klimešová J. Evolution of clonal growth forms in angiosperms. New Phytol 2020;225:999–1010. 10.1111/nph.16188 31505049

[plag028-B43] Hesp PA . Ecological processes and plant adaptations on coastal dunes. J Arid Environ 1991;21:165–91. 10.1016/S0140-1963(18)30681-5

[plag028-B44] Hesp PA . Foredunes and blowouts: initiation, geomorphology and dynamics. Geomorphology 2002;48:245–68. 10.1016/S0169-555X(02)00184-8

[plag028-B45] Hunziker JH . Cytogenetics and evolution of some species of the genus *Poa* (Gramineae). In: Drets Brum-Zorrilla ME, Folle GA (eds.), Actas III Congreso Latinoamericano de Genética. Montevideo: Asociación Latinoamericana de Genética, 1978, 144–9.

[plag028-B46] Huson DH, Bryant D. Application of phylogenetic networks in evolutionary studies. Mol Biol Evol 2006;23:254–67. 10.1093/molbev/msj030 16221896

[plag028-B46a] Index Herbariorum. *Index Herbariorum: A Global Directory of Public Herbaria and Associated Staff*. New York Botanical Garden, updated continuously. https://sweetgum.nybg.org/science/ih/ (8 June 2026, date last accessed).

[plag028-B47] Isla FI, Bértola G, Fernández JM et al Beach-morphodynamic changes conditioned by the Holocene sea-level fluctuation: Mesotidal beaches of Northern Patagonia. Acta Geológica Lilloana 2023;34:111–28. 10.30550/j.agl/2023.34.2/1830

[plag028-B48] Isla FI, Cortizo LC. Coastal erosion in Argentina: the retreating rates of southern South America. J South Am Earth Sci 2023;126:104342. 10.1016/j.jsames.2023.104342

[plag028-B49] Isla FI, Cortizo LC, Turno Orellano HA. Dinámica y evolución de las barreras medanosas, Provincia de Buenos Aires, Argentina. Rev Bras Geomorfol 2001;2:73–83. 10.20502/rbg.v2i1.9

[plag028-B50] Isla FI, Espinosa MA. Coastal environmental changes associated with Holocene sea-level fluctuation: Southeastern Buenos Aires, Argentina. Quat Int 1995;26:55–60. 10.1016/1040-6182(94)00046-8

[plag028-B51] Isla FI, Quiroz Londoño OM, Cortizo LC. Groundwater characteristics within loessic deposits: the coastal springs of Los Acantilados, Mar del Plata, Argentina. Environ Earth Sci 2018;77:610. 10.1007/s12665-018-7766-y

[plag028-B52] Islam MM, Deepo DM, Nasif SO et al Cytogenetics and consequences of polyploidization on different biotic-abiotic stress tolerance and the potential mechanisms involved. Plants 2022;11:2684. 10.3390/plants11202684 36297708 PMC9609754

[plag028-B53] IUCN . IUCN Red List Categories and Criteria: Version 3.1, 2nd edn. Gland, Switzerland and Cambridge, UK: IUCN, 2012, iv + 32pp.

[plag028-B54] Jombart T . Adegenet: a R package for the multivariate analysis of genetic markers. Bioinformatics 2008;24:1403–5. 10.1093/bioinformatics/btn129 18397895

[plag028-B55] Jombart T, Devillard S, Balloux F. Discriminant analysis of principal components: a new method for the analysis of genetically structured populations. BMC Genet 2010;11:94. 10.1186/1471-2156-11-94 20950446 PMC2973851

[plag028-B56] Joshi A, Shaun Bushman B, Pickett B et al Phylogenetic relationships among low-ploidy species of Poa using chloroplast sequences. Genome 2017;60:384–92. 10.1139/gen-2016-0110 28177839

[plag028-B57] Levin DA . The ecological transition in speciation. New Phytol 2004;161:91–6. 10.1046/j.1469-8137.2003.00921.x

[plag028-B58] Lewis SE, Wüst RA, Webster JM et al Mid-late Holocene sea-level variability in eastern Australia. Terra Nova 2008;20:74–81. 10.1111/j.1365-3121.2007.00789.x

[plag028-B59] Lima LG, Parise CK. Holocene coastal evolution of the transition from transgressive to regressive barrier in southern Brazil. CATENA 2020;185:104263. 10.1016/j.catena.2019.104263

[plag028-B60] López RA, Marcomini SC (eds.) Problemática de los ambientes costeros sur de Brasil, Uruguay y Argentina. Ed. Croquis, 2011, 211.

[plag028-B61] Malinsky M, Matschiner M, Svardal H. Dsuite—fast D-statistics and related admixture evidence from VCF files. Mol Ecol Resour 2021;21:584–95. 10.1111/1755-0998.13265 33012121 PMC7116594

[plag028-B62] Malinsky M, Svardal H, Tyers AM et al Whole-genome sequences of Malawi cichlids reveal multiple radiations interconnected by gene flow. Nat Ecol Evol 2018;2:1940–55. 10.1038/s41559-018-0717-x 30455444 PMC6443041

[plag028-B63] Marcomini S, López R, Picca P et al Natural coastal dune-field landforms, plant communities, and human intervention along Buenos Aires Northern Aeolian Barrier. J Coast Res 2017;335:1051–64. 10.2112/JCOASTRES-D-15-00219.1

[plag028-B64] Marcomini SC . Morfodinámica, sedimentología, geomorfología ambiental y sus alteraciones antropogénicas en costas de dunas del noreste de la provincia de Buenos Aires. Doctoral Thesis. Facultad de Ciencias Exactas y Naturales, Universidad de Buenos Aires, 2003, 360.

[plag028-B65] Monserrat AL, Celsi CE, Fontana S. Coastal dune vegetation of the southern Pampas (Buenos Aires, Argentina) and its value for conservation. J Coast Res 2012;28:23–35. 10.2307/41331986

[plag028-B66] Nei M, Kumar S. Molecular Evolution and Phylogenetics. Oxford University Press, 2000, 352.

[plag028-B67] Nosov NN, Punina EO, Machs EM et al Interspecies hybridization in the origin of plant species: cases in the genus *Poa* sensu lato. Biol Bull Rev 2015;5:366–82. 10.1134/S2079086415040088

[plag028-B68] Parodi LR . Una nueva especie de *Poa* de la flora Argentina. Notas del Museo de La Plata 1940;5:325–30.

[plag028-B69] Pembleton LW, Cogan NOI, Forster JW. StAMPP: an R package for calculation of genetic differentiation and structure of mixed-ploidy level populations. Mol Ecol Resour 2013;13:946–52. 10.1111/1755-0998.12129 23738873

[plag028-B70] Peng JC, He Z, Zhang ZQ. Standing genetic variation and introgression shape the cryptic radiation of Aquilegia in the mountains of Southwest China. Commun Biol 2025;8:684. 10.1038/s42003-025-08120-w 40307563 PMC12043930

[plag028-B71] Ponce JF, Rabassa J, Coronato A et al Palaeogeographical evolution of the Atlantic coast of Pampa and Patagonia from the last glacial maximum to the Middle Holocene. Biol J Linn Soc Lond 2011;103:363–79. 10.1111/j.1095-8312.2011.01653.x

[plag028-B72] Price MN, Dehal PS, Arkin AP. FastTree 2—approximately maximum-likelihood trees for large alignments. PLoS One 2010;5:e9490. 10.1371/journal.pone.0009490 20224823 PMC2835736

[plag028-B73] Pritchard JK, Stephens M, Donnelly P. Inference of population structure using multilocus genotype data. Genetics 2000;155:945–59. 10.1093/genetics/155.2.945 10835412 PMC1461096

[plag028-B74] Rieseberg LH, Brouillet L. Are many plant species paraphyletic? Taxon 1994;43:21–32. 10.2307/1223457

[plag028-B75] Rivera BJ, Belone J, Mathew AR et al How you dune-ing? A systematic review of coastal dune plant community assembly. J Coast Conserv 2025;29:23. 10.1007/s11852-025-01107-z

[plag028-B76] Sabena FR . Análisis de la variablidad morfológica de Poa bergii y P. lanuginosa y su relación con hospedantes de endofitos del género Epichloë. Bachelor Thesis. Facultad de Ciencias Exactas y Naturales, Universidad de Buenos Aires, 2019, 87.

[plag028-B77] Sassone AB, Blattner F, Giussani LM et al First glimpse of spring starflower domestication. Genes 2022;13:243. 10.3390/genes13020243 35205288 PMC8872604

[plag028-B78] Sassone AB, Hosjsgaard DH, Giussani LM et al Genomic, karyological and morphological changes of South American garlics (*Ipheion*) provide insights into mechanisms of speciation in the Pampean region. Mol Ecol 2021;30:3716–29. 10.1111/mec.16009 34087027

[plag028-B79] Saura F . Cariología de Gramíneas en Argentina. Revista de la Facultad de Agronomía y Veterinaria (Buenos Aires) 1948;12:51–67.

[plag028-B80] Scherer M, Marques Martins E, Silva T et al *Sand Dunes System of Southern South America. Red List of Ecosystems Assessment*. 2020. https://cm.iucnrle.org/assets/89ba67bf-c18f-4ed4-b0a6-c4656c393356 (20 November 2025, date last accessed).

[plag028-B81] Scrivanti LR, Mestre L, Anton AM. Phenotypical variation and taxonomic correlates of five closely related Andean species of *Poa* (Poaceae) along geographic and climatic gradients. Phytotaxa 2014;183:121–44. 10.11646/phytotaxa.183.3.1

[plag028-B82] Silva-Arias GA, Caballero-Villalobos L, Giudicelli GC et al Landscape and climatic features drive genetic differentiation processes in a South American coastal plant. BMC Ecol Evol 2021;21:196. 10.1186/s12862-021-01916-4 34702161 PMC8547116

[plag028-B83] Sloss CR, Shepherd M, Hesp P. Coastal dunes: geomorphology. Nat Educ Knowl 2012;3:2. https://www.nature.com/scitable/knowledge/library/coastal-dunes-geomorphology-25822000/

[plag028-B84] Sokal RR, Crovello TJ. The biological species concept: a critical evaluation. Am Nat 1970;104:127–53. 10.1086/282646

[plag028-B85] Soltis DE, Visger CJ, Soltis PS. The polyploidy revolution then…and now: Stebbins revisited. Am J Bot 2014;101:1057–78. 10.3732/ajb.1400178 25049267

[plag028-B86] Soltis PS, Marchant DB, Van de Peer Y et al Polyploidy and genome evolution in plants. Curr Opin Genet Dev 2015;35:119–25. 10.1016/j.gde.2015.11.003 26656231

[plag028-B87] Soltis PS, Soltis DE. The role of hybridization in plant speciation. Annu Rev Plant Biol 2009;60:561–88. 10.1146/annurev.arplant.043008.092039 19575590

[plag028-B88] Soltis PS, Soltis DE. Ancient WGD events as drivers of key innovations in angiosperms. Curr Opin Plant Biol 2016;30:159–65. 10.1016/j.pbi.2016.03.015 27064530

[plag028-B89] Soreng RJ . *Poa* L. In: Barkworth ME, Capels KM, Long SL, Anderton LK, Piep MB (eds.). Magnoliophyta: Commelinideae (in Part); Poaceae, Part 1, Flora of North America North of Mexico, 24. New York: Oxford University Press, 2007, 486–601.

[plag028-B90] Soreng RJ, Bull RD, Gillespie LJ. Phylogeny and reticulation in *Poa* based on plastid *trn*TLF and nrITS sequences with attention to diploids. In: Seberg O, Peterson G, Barfod A, Davis JI (eds.), Diversity, Phylogeny, and Evolution in the Monocotyledons. Aarhus: Aarhus University Press, 2010, 619–43.

[plag028-B91] Stebbins GL . Variation and Evolution in Plants. New York and London: Columbia University Press, 1950, 643.

[plag028-B92] Stull GW, Pham KK, Soltis PS et al Deep reticulation: the long legacy of hybridization in vascular plant evolution. Plant J 2023;114:743–66. 10.1111/tpj.16142 36775995

[plag028-B93] Toffani M, Hesp PA, Isla FI et al Evolutionary stages of active to vegetated coastal transgressive dunefields in the San Matías Gulf coast, Argentina. Geomorphology 2024;461:109289. 10.1016/j.geomorph.2024.109289

[plag028-B94] Torres MA . *Poa* L. In: Cabrera AL (ed.), Flora de la Provincia de Buenos Aires, Vol. 4. Buenos Aires: Colección Científica INTA, 1970, 102–5.

[plag028-B95] Tossi VE, Martínez Tosar LJ, Laino LE et al Impact of polyploidy on plant tolerance to abiotic and biotic stresses. Front Plant Sci 2022;13:869423. 10.3389/fpls.2022.869423 36072313 PMC9441891

[plag028-B96] Unmack PJ, Berry OF, Georges A et al DARTR: an R package to facilitate analysis of SNP data generated from reduced representation genome sequencing. Mol Ecol Resour 2018;18:691–9. 10.1111/1755-0998.12745 29266847

[plag028-B97] Van de Peer Y, Maere S, Meyer A. The evolutionary significance of ancient genome duplications. Nat Rev Genet 2009;10:725–32. 10.1038/nrg2600 19652647

[plag028-B98] Walker JM, van der Heijden Eva SM, Maulana A et al Common misconceptions of speciation. Evol J Linn Soc 2024;3:kzae029. 10.1093/evolinnean/kzae029 39600713 PMC11590199

[plag028-B99] Weir BS, Cockerham CC. Estimating F-statistics for the analysis of population structure. Evolution 1984;38:1358–70. 10.1111/j.1558-5646.1984.tb05657.x 28563791

[plag028-B100] Wendler N, Mascher M, Nöh C et al Unlocking the secondary gene-pool of barley with next-generation sequencing. Plant Biotechnol J 2014;12:1122–31. 10.1111/pbi.12219 25040223

[plag028-B101] Wu D, Mao K. Advancing plant phylogenetics and evolution through genomic data bursts and methodological innovations. J Syst Evol 2025;63:781–7. 10.1111/jse.70005

