# Supplementary figures and images for: Dioecious Poa species diversification parallels dune formation in Atlantic coastal ecosystems
Source: AoB Plants. 2026 Jun 8;18(4):plag028. doi: 10.1093/aobpla/plag028 (PMC13322398; doi:10.1093/aobpla/plag028)

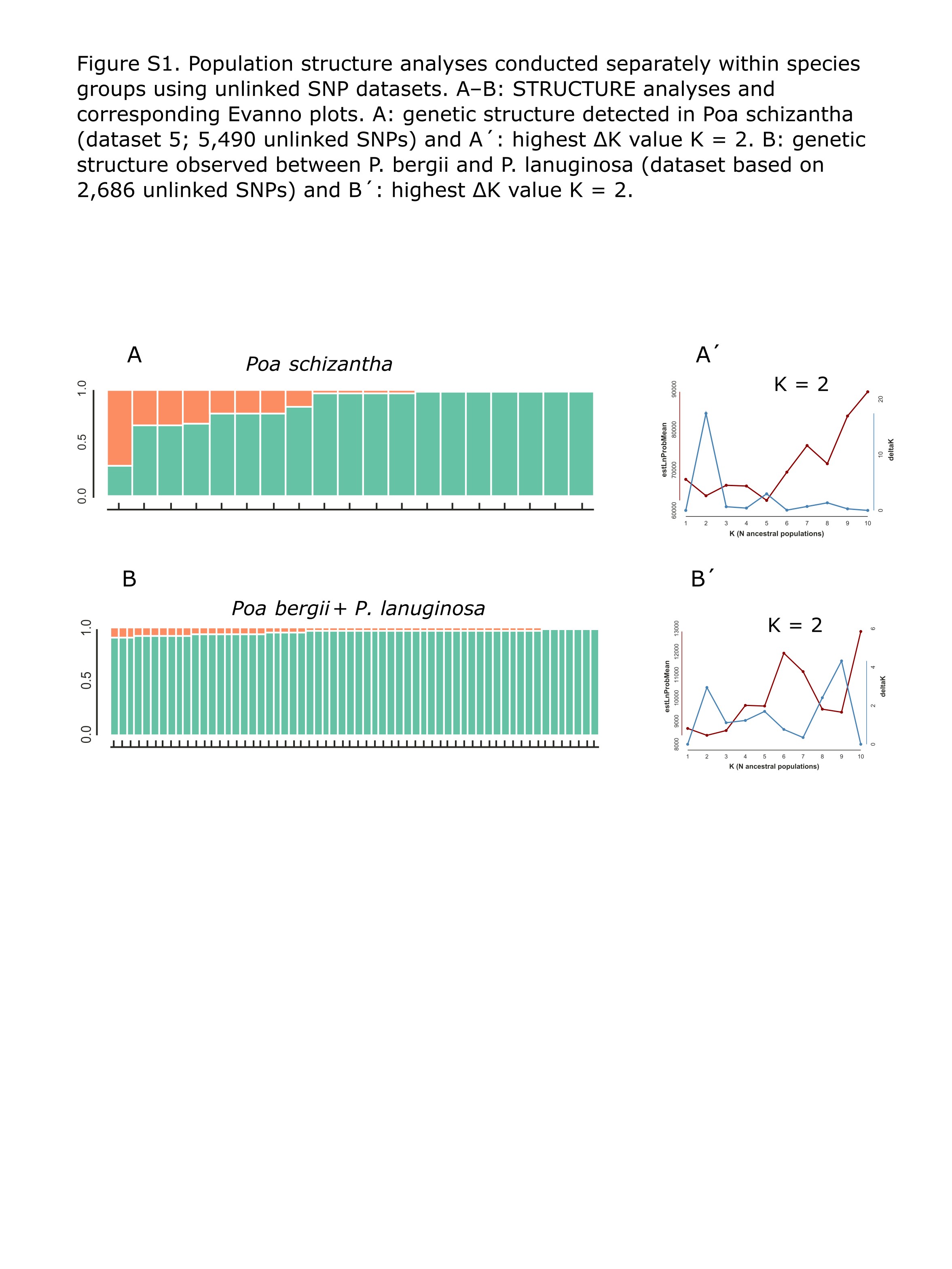

Supplement: plag028_Supplementary_Data [file plag028_supplementary_data.zip › Supplementary Figure S1_.jpg]
